# *unfulfilled* Interacting Genes Display Branch-Specific Roles in the Development of Mushroom Body Axons in *Drosophila melanogaster*

**DOI:** 10.1534/g3.113.009829

**Published:** 2014-02-20

**Authors:** Karen E. Bates, Carl Sung, Liam Hilson, Steven Robinow

**Affiliations:** Department of Biology, University of Hawaii, Honolulu, Hawaii 96822

**Keywords:** dHR51, CG16801, nuclear receptor, neuronal differentiation, suppressor screen

## Abstract

The mushroom body (MB) of *Drosophila melanogaster* is an organized collection of interneurons that is required for learning and memory. Each of the three subtypes of MB neurons, γ, α´/β´, and α/β, branch at some point during their development, providing an excellent model in which to study the genetic regulation of axon branching. Given the sequential birth order and the unique patterning of MB neurons, it is likely that specific gene cascades are required for the different guidance events that form the characteristic lobes of the MB. The nuclear receptor UNFULFILLED (UNF), a transcription factor, is required for the differentiation of all MB neurons. We have developed and used a classical genetic suppressor screen that takes advantage of the fact that ectopic expression of *unf* causes lethality to identify candidate genes that act downstream of UNF. We hypothesized that reducing the copy number of *unf*-interacting genes will suppress the *unf*-induced lethality. We have identified 19 candidate genes that when mutated suppress the *unf*-induced lethality. To test whether candidate genes impact MB development, we performed a secondary phenotypic screen in which the morphologies of the MBs in animals heterozygous for *unf* and a specific candidate gene were analyzed. Medial MB lobes were thin, missing, or misguided dorsally in five double heterozygote combinations (*;unf/+;axin/+*, *unf/+;Fps85D/+*, *;unf/+;Tsc1/+*, *;unf/+;Rheb/+*, *;unf/+;msn/+*). Dorsal MB lobes were missing in *;unf/+;DopR2/+* or misprojecting beyond the termination point in *;unf/+;Sytβ* double heterozygotes. These data suggest that *unf* and *unf*-interacting genes play specific roles in axon development in a branch-specific manner.

A complex axonal branching pattern of interneurons allows single neurons to signal multiple downstream target neurons. Current models for the formation of a branched axon include growth cone splitting or the formation of a collateral from the axonal shaft and require that at some point a single axon of a single cell must pathfind simultaneously or serially to two or more different targets (Gibson and Ma 2011; Lewis *et al.* 2013; Schmidt and Rathjen 2010). The mushroom body (MB) of *Drosophila melanogaster* provides an excellent system in which to investigate the genetic regulation of axon branching because all MB axons form two branches at some point during their development.

The *Drosophila* MB is an ordered structure that is the learning center of the fly brain (Davis 2005; Zars 2000). Each of the three subtypes of MB neurons, the γ, α´/β´, and α/β neurons, follows a distinct developmental program (Armstrong *et al.* 1998; Lee *et al.* 1999; Technau and Heisenberg 1982). The γ neurons are the first to extend axons anteroventrally, forming the peduncle, a thick bundle of fasciculated axons. The axons reach a choicepoint where they first project medially forming the medial lobe. Formation of the dorsal lobe follows as a result of collateral branching (Kurusu *et al.* 2002). During metamorphosis, these γ axons are pruned back into the peduncle and then re-extend medially only. Prior to γ axon pruning, the second-born α´/β´ neurons grow along the existing peduncle until they reach the same choicepoint. These α´/β´ neurons extend axons both medially and dorsally. The last-born α/β neurons also project axons both medially and dorsally and like the γ and α´/β´ neurons, form their own distinct lobes. In contrast to the γ neurons, the branching of these later-born neurons may be a result of growth cone splitting rather than collateral formation (Wang *et al.* 2002). Given the sequential birth order and the formation of five MB lobes, it is conceivable that distinct genetic programs govern the development of these distinct populations of MB neurons.

During MB development the transcription factor UNFUFILLED (UNF) is required for axon pathfinding beyond the choicepoint for all three subtypes of MB neurons (Bates *et al.* 2010). Indirect data support the hypothesis that UNF acts as a transcriptional repressor (Palanker *et al.* 2006; Yaniv *et al.* 2012). However, the extensive data showing that PNR, the vertebrate ortholog of *unf*, functions both as an activator and repressor supports the hypothesis that UNF also acts as both a transcriptional activator and repressor of target genes (Chen *et al.* 2004, 2005; Haider *et al.* 2009). Identification of these target and downstream genes may shed light on the genetic regulation of branch formation.

To identify *unf*-dependent genes, we conducted a classic suppressor screen. Enhancer/suppressor screens in *Drosophila* have been particularly successful in identifying interacting loci (Casso *et al.* 2008; Ma *et al.* 2009; Sousa-Guimaraes *et al.* 2011). This suppressor screen takes advantage of the fact that 100% of animals in which the *OK107-GAL4* enhancer trap transgene drives the expression of a *UAS-unf* transgene develop to late pupal stages but fail to eclose (die as late-stage pupae). We hypothesized that if UNF is activating target genes that are causing this lethality, then removing one copy of an UNF target gene in this background (*;;UAS-unf;OK107-GAL4*) might suppress the lethal phenotype. Nineteen candidate genes were identified that suppressed the *OK107 > unf*-induced lethality. We then performed a secondary phenotypic screen in which the MBs of animals heterozygous for *unf* and heterozygous for a candidate gene were analyzed. MB defects were observed in seven double heterozygote combinations. The defects observed demonstrate that *unf*-interacting genes regulate MB development in a branch-specific manner.

## Materials and Methods

### Genetics

Third chromosome deficiencies, *OK107-GAL4*, *Ilp2-GAL4*, and stocks carrying mutations in candidate genes were obtained from Bloomington *Drosophila* Stock Center (flystocks.bio.indiana.edu; see Supporting Information, File S1). The *;FRTG13UAS-mCD8*::*GFP;;OK107-GAL4* (referred to as *;UAS-mCD8;;OK107*) line was a gift from L. Luo (Stanford University). The *;unf^X1^*/CyO and *;unf^X1^FRTG13UAS-mCD8*::*GFP/CyO;;OK107-GAL4* (referred to as *;unf^X1^UAS-mCD8;;OK107*) mutant lines and the *;;UAS-unfF1* transgenic line were generated in the Robinow lab (Sung *et al.* 2009). Double heterozygote tests were performed by crossing *;unf^X1^/CyO* or *;unf^X1^FRTG13UAS-mCD8/CyO;;OK107* heterozygotes to homo- or heterozygous mutants of candidate genes. Flies were raised on standard cornmeal and sugar medium at 25° with the exception of the suppressor screen, which was conducted at 22°.

Several controls were performed prior to beginning the initial suppressor screen. All flies carrying both the *OK107-GAL4* and *UAS-unf* transgenes develop to late pupal stages but fail to eclose (Bates *et al.* 2010). These dead pupae have small or no eyes, almost certainly due to *OK107-GAL4*-driven expression of *unf* in the developing visual system. In contrast, flies containing the *OK107-GAL4* and *UAS-mCD8* transgenes develop and eclose normally. Since GAL4 activity is temperature-dependent (Duffy 2002), *;;UAS-unfF1* virgins were crossed to *;;;OK107-GAL4* males and raised at 25°, 22°, or 20° to test whether temperature had an effect on *OK107*>*unf*-induced lethality. When performed at 25° or 22°, small-eyed flies were never observed in any of three vials of independent crosses. When raised at 20°, one small-eyed survivor was collected from one of three vials. Suppression of the *OK107 > unf*-induced lethality was determined by the presence of any small-eyed flies. Both the number of small-eyed flies and the number of siblings of all other possible genotypes (*n*) are reported in Table 1. Initially, sibling flies were not individually scored, and instead only vials were counted. In these cases *n* is only approximate and is based on the observation that each of the scored vials contained approximately 50 pupae.

**Table 1:** Suppression of lethality induced by ectopic expression of *unfulfilled* (*unf*)

Row	Deficiency/Mutant	Start Break-Points	End Break-Points	Small Eye Flies (n)	Candidate Genes
1	*Df(3L)ED50002*	61A1	61B1	0 (31)	
2	*Df(3L)ED201*	61B1	61C1	4 (61)	*Ptpmeg*
3	*Df(3L)BSC362*	61C1	61C7	2 (61)	*Ptpmeg*
4	*Df(3L)ED4177*	61C1	61E2	0 (45)	*Ptpmeg*
5	*Ptpmeg^1^*	61C1	61C1	0 (29)	
6	*Df(3L)BSC289*	61F6	62A9	0 (46)	
7	*Df(3L)BSC181*	62A11	62B7	1 (70)	*a-Spec*, *dlt*
8	*Df(3L)Aprt-32*	62B1	62E3	1 (113)	*a-Spec*, *dlt*, *msn*
9	*Df(3L)ED4287*	62B4	62E5	2 (165)	*a-Spec*, *dlt*, *msn*
10	*Df(3L)BSC119*	62E7	62F5	6 (61)	*msn*
11	*Df(3L)M21*	62F	63D	5 (113)	*msn*, *spz5*, *Shab*, *gry*
12	*Df(3L)Exel6092*	62F5	63A3	2 (39)	*spz5*
13	*Df(3L)BSC672*	63A7	63B12	1 (87)	*gry*
14	*Df(3L)ED4293*	63C1	63C1	5 (111)	
15	*Df(3L)ED208*	63C1	63F5	2 (44)	
16	*Df(3L)BSC368*	63F1	64A4	0 (91)	
17	*a-Spec^lm88^*	62B4	62B4	0 (39)	
18	dlt^04276^,a-spec^04276^*	62B4	62B4	17 (26)	
19	msn^102^*	62E6	62E7	1 (29)	
20	*spz5^E03444^*	63A1	63A1	0 (42)	
21	Shab^MB02726^*	63A1	63A2	2 (33)	
22	*gry^EY03013^*	63B11	63B13	0 (48)	
23	*Df(3L)ED210*	64B9	64C13	0 (167)	*Klp64D*
24	*Df(3L)ZN47*	64C	65C	1 (32)	*Klp64D*, *S6K*, *dikar*, *velo*
25	*Df(3L)BSC371*	64C1	64E1	3 (66)	*Klp64D*
26	*Df(3L)BSC410*	64E7	65B3	4 (85)	*S6K*
27	*Df(3L)Exel6109*	65C3	65D3	5 (58)	*dikar*, *velo*
28	*Df(3L)BSC224*	65D5	65E6	2 (64)	*sgl*
29	*Df(3L)Exel8104*	65F7	66A4	0 (37)	
30	*Klp64D^K1^*	64C13	64C13	0 (41)	
31	*S6K^l-1^*	64E8	64E11	0 (27)	
32	*dikar^d02315^*	65C3	65C3	0 (34)	
33	*velo^EY10127^*	65C3	65C3	0 (48)	
34	*sgl^08310^*	65D4	65D5	0 (40)	
35	*Df(3L)BSC117*	65E9	65F5	1 (16)	
36	*Df(3L)BSC375*	66A3	66A19	0 (23)	
37	*Df(3L)BSC388*	66A8	66B11	2 (67)	*Arp3*
38	*Df(3L)Exel6112*	66B5	66C8	2 (71)	*Arp3*
39	*Df(3L)BSC815*	66C3	66D4	0 (39)	
40	*Arp3^EP3640^*	66B6	66B6	0 (33)	
41	*Df (3L)BSC816*	66D9	66D12	1 (33)	
42	*Df(3L)ED4421*	66D12	67B3	0 (43)	
43	*Df(3L)BSC113*	67B1	67B5	2 (40)	*aay*
44	*Df(3L)BSC391*	67B7	67C5	1 (53)	
45	*Df(3L)BSC392*	67C4	67D1	4 (42)	*a-Tub67C*, *GAP1*
46	*Df(3L)BSC673*	67C7	67D10	4 (61)	*a-Tub67C*, *GAP1*
47	*Df(3L)ED4457*	67E2	68A7	0 (4)	
48	aay^S042314^*	67B5	67B5	9 (33)	
49	*GAP1^B2^*	67C10	67C11	0 (27)	
50	a−Tub67C^1^*	67C4	67C4	2 (46)	
51	*Df(3L)4486*	6974	69F6	0 (77)	
52	*Df(3L)BSC12*	69F6-70A1	70A1-2	2 (30)	*trn*
53	*Df(3L)ED4502*	70A3	70C10	3 (40)	*caps*
54	*Df(3L)ED4543*	70C6	70F4	0 (131)	
55	*trn^S064117^*	70A1	70A1	0 (29)	
56	caps^02937^*	70A3	70A4	2 (33)	
57	*Df(3L)ED4543*	70C6	70F4	0 (131)	
58	*Df(3L)ED217*	70F4	71E1	1 (65)	*Sytβ*
59	*Df(3L)BSC845*	71D3	72A1	3 (63)	*comm*
60	*Df(3L)BSC774*	71F1	72D10	0 (39)	*comm*
61	Sytβ^PL00192^**	71B2	71B2	2 (16)	
62	*Sytβ^BG02150^*	71B2	71B2	0 (46)	
63	*comm^MI00380^*	71F2	71F2	0 (26)	
64	*Df(3L)BSC774*	71F1	72D10	0 (39)	
65	*Df(3L)ED220*	72D4	72F1	6 (50)*^c^*	*fax*
66	*Df(3L)ED4606*	72D4	73C4	7 (100)*^c^*	*fax*, *Abl*
67	*Df(3L)BSC555*	72E2	73A10	17 (50)*^c^*	*fax*
68	*Df(3L)ED223*	73A1	73D5	2 (50)*^c^*	*Abl*
69	*Df(3L)81k19**^a^*^,^*^b^*	73A3	74F1-74F4	4 (50)*^c^*	*Abl*
70	*Df(3L)ED4674*	73B5	73E5	1 (100)*^c^*	
71	*Df(3L)ED4685*	73D5	74E2	0 (50)*^c^*	
72	fax^M7^*	72E5	72F1	36 (150)*^c^*	
73	fax^BG00833^*	72E5	72F1	18 (100)*^c^*	
74	fax^EY01882^*	72E5	72F1	13 (50)*^c^*	
75	fax^KG05016^*	72E5	72F1	19 (50)*^c^*	
76	*Abl^2^*	73B1	73B4	0 (34)	
77	*Df(3L)BSC20*	76A7-B1	76B4-B5	3 (91)	
78	*Df(3L)BSC797*	77C3	78A1	0 (14)	
79	*Df(3L)BSC449*	77F2	78C2	1 (49)	*siz*, *chb*
80	*Df(3L)BSC553*	78A2	78C2	1 (26)	*siz*, *chb,*
81	*Df(3L)BSC419*	78C2	78D8	0 (31)	*chb*
82	*siz^EY09677^*	78A5	78B1	0 (36)	
83	*chb^4^*	78C1	78C2	0 (56)	
84	*Df(3L)BSC419*	78C2	78D8	0 (31)	
85	*Df(3L)ED4978*	78D5	79A2	1 (50)	*mub*
86	*Df(3L)BSC223*	79A3	79B3	6 (39)	*mub*
87	*Df(3L)BSC451*	79B2	79F5	3 (58)	*Ten-m*
88	*Df(3L)ED230*	79C2	80A4	1 (11)	*Ten-m*
89	*Df(3L)ED5017*	80A4	80C2	2 (76)	
90	*Df(3L)1-16*	80F	80F	0 (73)	
91	*mub^04093^*	78F4	79A3	0 (58)	
92	Ten-m^05309^*	79D4	79E3	5 (53)	
93	*Df(3R)ED5156*	82F8	83A4	0 (47)	
94	*Df(3R)BSC549*	83A6	83B6	4 (34)	*Nmdar1*, *Rheb*
95	*Df(3R)Exel6144*	83A6	83B6	1 (78)	*Nmdar1*, *Rheb*
96	*Df(3R)BSC464*	83B7	83E1	2 (45)	*Nmdar1*, *Rheb*
97	*Df(3R)BSC681*	83E2	83E5	0 (36)	
98	*Nmdar^105616^*	83A6	83A7	0 (65)	
99	Rheb^EY08085^*	83B2	83B2	4 (15)	
100	*Df(3R)BSC507*	85D6	85D15	1 (32)	*Fps85D*
101	Fps85D^X21^**	85D13	85D15	7 (27)	
102	*Df(3R)BSC568*	86C7	86D7	2 (65)	
103	*Df(3R)BSC741*	88E8	88F1	3 (66)	*Tm1*, *Sra1*
104	*Tm1^02299^*	88E12	88E13	0 (40)	
105	*Sra1^EY06562^*	88F1	88F1	0 (47)	
106	*Df(3R)BSC515*	88F6	89A8	0 (31)	*Sap47*
107	*Df(3R)Exel7327*	89A8	89B1	1 (25)	*Sap47*
108	*Df(3R)BSC728*	89A8	89B2	10 (54)	*Sap47*
109	*Df(3R)Exel7328*	89A12	89B6	0 (23)	
110	Sap47^EY07944^*	89A8	89A8	4 (27)	
111	*Df(3R)Exel7328*	89A12	89B6	0 (23)	
112	*Df(3R)BSC887*	89B6	89B16	3 (116)	*gish*
113	*Df(3R)ED10639*	89B7	89B18	2 (54)	*gish*
114	*Df(3R)Exel6269*	89B12	89B18	4 (62)	*gish*
115	*Df(3R)ED10642*	89B17	89D5	0 (27)	
116	gish^KG03891^*	89B9	89B12	1 (61)	
117	*Df(3R)BSC748*	89E5	89E11	4 (89)	*dad*
118	*dad^J1E4^*	89E11	89E11	0 (17)	
119	*Df(3R)BSC619*	94D10	94E13	3 (51)	*hh*
120	*hh^2^*	94E1	94E1	0 (37)	
121	*Df(3R)ED6187*	95D10	96A7	0 (43)	*Tsc1*, *Syx1A*, *jar*
122	*Df(3R)Exel6198*	95E1	95F8	5 (118)	*Tsc1*, *Syx1A*, *jar*
123	*Dfslo3^b^*	95E7	96A18	6 (42)	*Syx18*, *slo*
124	*Df(3R)BSC317*	95F2	95F11	2 (89)	
125	*Df(3R)Exel6199*	95F8	96A2	7 (179)	*jar*
126	*Df(3R)Exel7357*	96A2	96A13	1 (46)	*Syx18*
127	*Df(3R)BSC397*	96A13	96A22	0 (33)	*Syx18*
128	Tsc1^F01910^*	95E1	95E1	2 (29)	
129	*Syx1A ^Δ229^*	95E1	95E1	0 (32)	
130	jar^1^*	95F6	95F8	6 (77)	
131	*Syx18^EY08095^*	96A12	96A13	0 (30)	
132	slo^1^*	96A14	96A17	7 (94)	
133	*Df(3R)BSC497*	97E6	98B5	0 (26)	
134	*Df(3R)ED6280*	98B6	98B6	4 (71)	
135	*Df(3R)BSC567*	98B6	98E5	1 (21)	
136	*Df(3R)BSC874*	98E1	99A1	1 (19)	
137	*Df(3R)BSC501*	98F10	99B9	0 (55)	*DopR2*
138	DopR2^MB05107^***	99B5	99B6	0 (42)	
139	*Df(3R)BSC620*	99C5	99D3	0 (89)	*axn*
140	*Df(3R)X3F^b^*	99D1-D2	99E1	7 (66)	*axn*
141	*Df(3R)BSC502*	99D3	99D8	1 (42)	*axn*
142	*Df(3R)Exel6214*	99D5	99E2	0 (76)	
143	axn^EY10228^**	99D2	99D3	6 (84)	
144	*Df(3R)BSC503*	99E3	99F6	3 (71)	
145	*Df(3R)BSC504*	99F4	100A2	0 (41)	
146	*Df(3R)A113^b^*	100A	100F	1 (50)*^c^*	*tll*, *dco*
147	*Df(3R)ED6346*	100A5	100B1	3 (29)	*tll*, *dco*
148	*Df(3R)BSC793^r^*	100B5	100C4	1 (59)	
149	*Df(3R)ED6361*	100C7	100E3	1 (49)	*ttk*
150	*Df(3R)BSC505*	100D1	11D2	0 (39)	*ttk*
151	tll^1^*	100A6	100A6	4 (49)	
152	tll^149^*	100A6	100A6	7 (63)	
153	dco^j3B9^*	100B1	100B2	2 (34)	
154	*ttk^1e11^*	100D1	100D1	0 (44)	

Notes: Suppression of the *OK107 > unf*-induced lethality was determined by the presence of any small-eyed flies. Both the number of small-eyed flies and the number of siblings of all other possible genotypes (*n*) are reported. MB, mushroom body.

* Deficiencies and candidates that suppress the *OK107 > unf*-induced lethality.

** Candidates that suppress the lethality and impact MB development in a secondary phenotypic screen.

*** Candidates that do not suppress the *OK107 > unf*-induced lethality but do impact MB development.

a First deficiency that produced small-eyed flies and subsequently used as positive control.

b Poorly defined deficiencies for which the breakpoints are only approximate.

c Approximate number of sibling flies (*n*), for cases in which vials instead of individual sibling flies were scored, is based on the observation that each of the scored vials contained approximately 50 pupae. Some overlapping deficiencies are reported in Table S1.

The efficacy of the *OK107 > unf*-induced lethality may also be modulated by the presence of additional UAS elements. When *;;UAS-unfF1* virgins were crossed to *;FRTG13UAS-mCD8;;OK107* males and raised at 25°, small-eyed flies were never observed, as expected. However, when raised at 22°, eight small-eyed flies were collected. These data suggest that the presence of the additional *UAS-mCD8* transgene, which may compete with the *UAS-unfF1* transgene for GAL4 activity, increases survivability by decreasing the expression of ectopically expressed *unf*. During the suppressor screen, certain crosses involved a *UAS-mCD8* element. In these situations, flies expressing this element were excluded from the analysis.

For the suppressor screen, F2 progeny were screened for small-eyed survivors. The small eye phenotype indicates that these flies carry both the *OK107-GAL4* transgene and one *UAS-unf* transgene. It is expected that 100% of these flies will be dead.
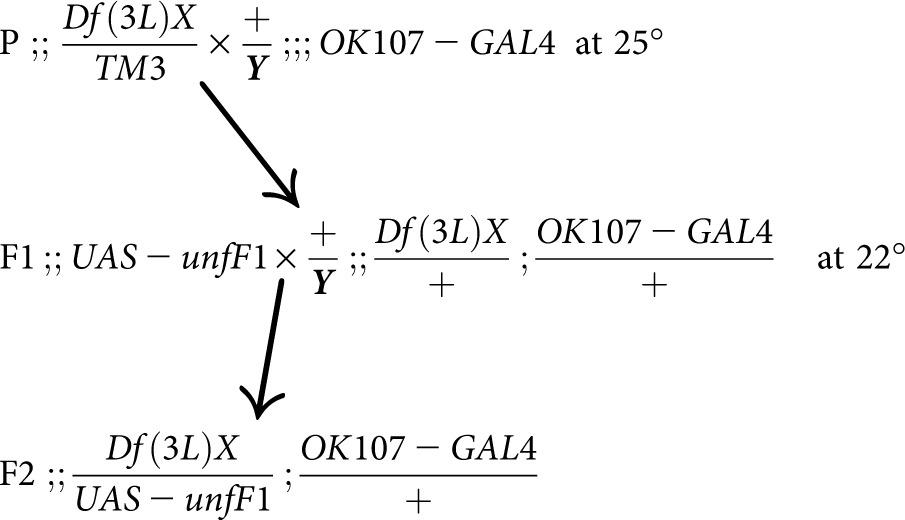
For a number of deficiency crosses, *;FRTG13UAS-mCD8;;OK107-GAL4* males were used instead of *;;;OK107-GAL4* males due to *;;;OK107-GAL4* being a particularly weak stock. In these cases, only small-eyed F2 progeny negative for GFP expression were scored. For negative controls, *;;UAS-unfF1* virgins were routinely crossed to *;;;OK107-GAL4/+* or *;FRTG13UAS-mCD8/+;;OK107-GAL4/+* males at 22° to continuously monitor and ensure the stringency of the screen.

### Immunohistochemistry and microscopy

Third instar larvae and 72- to 120-hr pupae were staged as described (Andres and Thummel 1994; Bainbridge and Bownes 1981). The nervous systems of pupae and 0- to 5-d-old adults were dissected, fixed in 4% paraformaldehyde, and processed using standard protocols (Lee and Luo 1999). mAb1D4 (Van Vactor *et al.* 1993) (anti-Fasciclin II; anti-Fas II; 1:10) and mAb9.4A (Awasaki *et al.* 2000) (anti-Trio; 1:4) were obtained from the Developmental Studies Hybridoma Bank. The rabbit anti-Fas II (1:3000) was a gift from Vivian Budnik (University of Massachusetts). The rabbit anti-crustacean cardioactive peptide (anti-CCAP; 1:10,000) was a gift from John Ewer (University of Valparaiso, Chilé). Biotinylated anti-mouse and anti-rabbit IgG (1:200) were obtained from Vector Labs (cat. No. BA-9200 and BA-1000, respectively). Streptavidin Alexa Fluor 488, 546, and 568 (1:200) were obtained from Invitrogen (cat. No. S11223, S11225, and S11226, respectively). Preparations were imaged by confocal laser scanning microscopy using a Zeiss LSM 710 confocal microscope. Images were processed using ImageJ 1.46j (National Institutes of Health) and Photoshop CS5, and InDesign CS5 (Adobe).

### Statistics

The Fisher’s exact test was used to determine whether the frequency of MB defects in experimental animals was significantly different from the frequency of defects in control animals. Relevant genotypes were tested in pair-wise combinations. One-tailed *p*-values less than 0.05 were considered significant. Because a significant effect could have been missed due to small sample sizes for each of the pair-wise combinations, the Fisher’s exact test was also used to determine whether the frequency of defects in experimental animals was significantly different from the frequency of defects in pooled control animals associated with a candidate gene and of the same genetic background, such as those with or without the *OK107-GAL4* and *UAS-mCD8* transgenes. This method allows us to report a *p*-value for the aggregated evidence across pair-wise combinations regardless of the significance of any individual test and allows us to regain some of the power lost by dividing the control data into smaller groups. A multiple comparison correction was not performed because the candidate genes were first identified as suppressors of the *OK107 > unf*-induced lethality.

## Results

### Characterization of lethality induced by ectopic expression of *unf*

**Figure 1:**
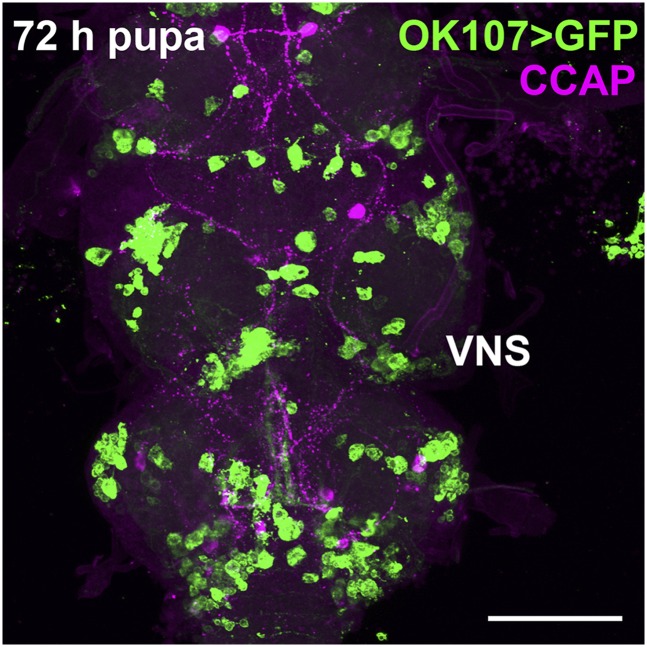
*OK107-GAL4* drives expression in the ventral nervous system (VNS). In this *;;UASmCD8GFP;;OK107-GAL4* 72-hr pupa labeled with anti-crustacean cardioactive peptide (CCAP), *OK107-GAL4*-driven GFP is expressed in heterogeneous cells throughout the VNS but not in the CCAP-expressing cells. Scale bar = 200 μm.

This suppressor screen takes advantage of the fact that 100% of animals in which the *OK107-GAL4* enhancer trap transgene drives the expression of a *UAS-unf* transgene develop to late pupal stages but fail to eclose. The inference is that the ectopic expression of the transcriptional regulator UNF has disrupted the function of a set of cells that are required for the latest stages of pupal development or eclosion. Our efforts to identify the cells responsible for this lethality have been unsuccessful. *OK107-GAL4* drives expression in the MB, optic lobes, antennal lobes, and the pars intercerebralis (Adachi *et al.* 2003; Aso *et al.* 2009; Connolly *et al.* 1996) and in a large uncharacterized set of ventral neurons (Figure 1). Since the MB, the eyes, and the antennal lobes are not required for viability (Callaerts *et al.* 2001; de Belle and Heisenberg 1994), the lethality almost certainly is due to expression in the pars intercerebralis or the uncharacterized ventral neurons. To test whether *unf* expression in the pars intercerebralis could be responsible for the pupal lethality observed in the *OK107 > unf* animals, we used an *Ilp2-GAL4* transgene to drive expression in a subset of pars intercerebralis neurons that express the insulin-like peptide 2 (Ilp2) (Rulifson *et al.* 2002). Expression of *unf* in the Ilp2 neurons results in a larval lethality. All *Ilp2 > unf* animals die as larvae, not pupae. These data suggest that the Ilp2 neurons of the pars intercerebralis are not responsible for the *OK107 > unf*-induced pupal lethality. Additional investigations using a variety of other drivers and cell markers, including anti-CCAP to label CCAP-expressing neurons in the brain and ventral nervous system, were not helpful in localizing the neurons responsible for the *OK107 > unf*-induced lethality (Figure 1).

### A suppressor screen to identify genomic regions that encode *unf*-interacting genes

**Figure 2:**
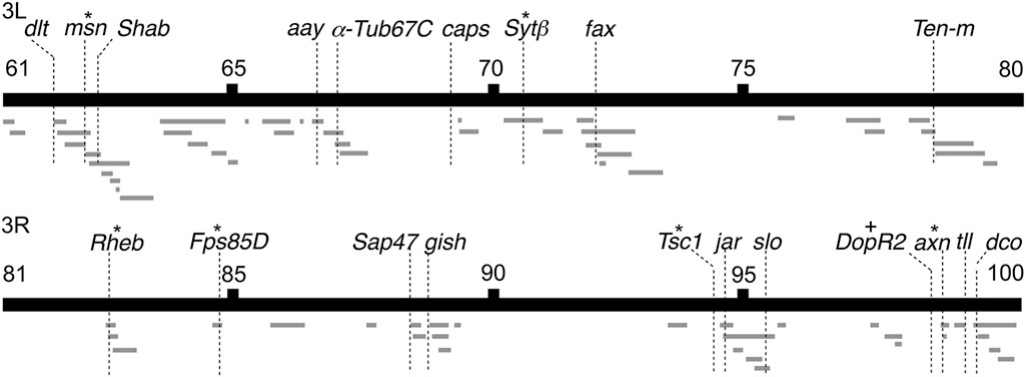
Suppressors of the *OK107 > unfulfilled* (*unf*)-induced lethality. This schematic maps the third chromosome deficiencies and the 19 candidate genes that suppress the *OK107 > unf*-induced lethality. *Candidate genes that suppress the lethality and impact mushroom body development in a secondary phenotypic screen. ^+^*DopR2* does not suppress the lethality but does impact mushroom body development. 3L, left arm; 3R, right arm. Not to scale.

This screen is based on the underlying assumption that the *OK107 > unf*-induced lethality is due to the *unf*-dependent activation of target genes and other indirectly regulated downstream genes. We hypothesized that reducing the copy number of one of these *unf*-dependent genes would suppress the *OK107 > unf*-induced lethality, resulting in the survival of some animals. Suppression of the *OK107 > unf*-induced lethality was determined by the presence of any small-eyed flies. Both the number of small-eyed flies and the number of siblings of all other possible genotypes (*n*) are reported in Table 1. Of the 177 third chromosome deficiencies that were tested, 103 deficiencies from 26 distinct regions suppressed the *OK107 > unf*-induced lethality (Table 1, Figure 2, and Table S1). To limit the region responsible for the suppression of lethality overlapping deficiencies were sometimes tested.

### The identification of genes responsible for the suppression of the *OK107 > unf*-induced lethality

Forty-five candidate genes were identified within 21 of the 26 regions that suppressed the *OK107 > unf*-induced lethality. Candidate genes were not identified in five of the regions that suppressed the *OK107 > unf*-induced lethality. We defined a candidate gene as one known to have a role in nervous system development or neural function and that resides within the boundaries of deficiencies that suppress the *OK107 > unf*-induced lethality. This screen was not designed to test every possible gene within a deficiency of interest. Instead, we made the strategic decision to pursue genes already known to have some function within the nervous system.

Mutant alleles of the 45 candidate genes were tested for their ability to suppress the *OK107 > unf*-induced lethality. Alleles that were tested were chosen based on previously reported neuronal phenotypes or the severity of the allele. Multiple alleles were tested when loss-of-function alleles were not available or when the available alleles were uncharacterized. Of the 45 genes tested, 19 candidate genes within 14 genomic regions suppressed this lethality (Table 1). None of 13 candidate genes distributed among seven genomic regions suppressed this lethality. Lastly, we were unable to identify any candidate genes in five regions that suppressed the *OK107 > unf*-induced lethality.

Beginning with the left arm of the third chromosome, nine overlapping deficiencies spanning the **62A11;63F5 region** suppressed the lethality. Candidate genes found in one or more of these deficiencies include *α-Spectrin (α-Spec^lm88^*) (Garbe and Bashaw 2007); *discs lost* (*dlt^04276^*; also known as *DPATJ*), which shares a first untranslated exon with *α-Spec* (Nam and Choi 2006; Pielage *et al.* 2003); *misshapen* (*msn^102^*) (Ruan *et al.* 1999; Su *et al.* 2000); *späetzal* (*spz5^E03444^*) (Zhu *et al.* 2008); *Shaker cognate b (Shab^MB02726^*) (Gasque *et al.* 2005); and *gryzun* (*gry^EY03013^*) (Akalal *et al.* 2011; Dubnau *et al.* 2003). Small-eyed flies were observed for two of the tested alleles, *msn^102^* and *Shab^MB02726^*. Because *dlt* single mutants were not available, *dlt^04276^ α-Spec^04276^* double mutants were tested and found to suppress the *OK107 > unf*-induced lethality. Single *α-Spec^lm88^* mutants did not, suggesting that the *dlt* mutation in the double mutant was responsible for the suppression (Table 1, Rows 17−22). Four deficiencies spanning the **7B1;67D10 region** suppressed the *OK107 > unf*- induced lethality. Of the three candidate genes in this region the alleles *astray* (*aay^S042314^*) (Salzberg *et al.* 1997) and *α-Tubulin67C* (*α-Tub67C^1^*) (Wang *et al.* 2007) suppressed the lethality, whereas *RasGAP1* (*GAP1^B2^*) (Yang and Terman 2012) did not (Table 1, Rows 48−50). In the **69F6;70C10 region**, two overlapping deficiencies suppressed the *OK107 > unf*-induced lethality. In this region, the *capricious* (*caps^02937^*) (Abrell and Jackle 2001) allele suppressed the lethality, but *tartan* (*trn^S064117^*) (Kurusu *et al.* 2008) did not (Table 1, Rows 55, 56). Two deficiencies in the **70F4;72A1 region** suppressed the *OK107 > unf*-induced lethality. Of the candidate genes that were tested, the *Synaptotagminß* (*Sytβ^PL00192^*) (Mackler and Reist 2001) allele suppressed the lethality, whereas *Sytβ^BG02150^* or *commissureless* (*comm^M100380^*) (Tear *et al.* 1996) did not (Table 1, Rows 61−63). This allele-specific suppression for *Sytβ* suggests that the *Sytβ^BG02150^* allele is a hypomorph and that the *Sytβ^PL00192^* allele is either a more severe hypomorph or an amorphic allele of *Sytβ*. The molecular nature of these alleles has not been determined.

*Df(3L)81k19* in the **72D4;74F4 region** was the first deficiency to be identified as a suppressor of the *OK107 > unf*-induced lethality based on the presence of four small-eyed flies at 22° (Table 1, Row 69). Crosses were performed at 25°, 22°, and 20° and compared with *;;UAS-unfF1/+;OK107-GAL4/+* negative controls. Six *;Df(3L)81k19/UAS-unfF1;OK107-GAL4/+* small-eyed flies were collected from one vial at 25°, four were collected from a total of two vials at 22°, and six were collected from a total of four vials at 20°. Due to its robust ability to suppress the *OK107 > unf*-induced lethality, *Df(3L)81k19* was used as a positive control with all subsequent crosses. Five other overlapping deficiencies spanning the region suppressed the *OK107 > unf*-induced lethality. *failed axon connections* (*fax*) and *Abl tyrosine kinase* (*Abl*) were identified as candidate genes based on their known cooperative roles in embryonic axon pathfinding (Hill *et al.* 1995; Liebl *et al.* 2000), and the observation that both lie within or near the breakpoints of suppressing deficiencies. We tested the ability of the *Abl^2^* allele and four *fax* alleles to suppress the *OK107 > unf*-induced lethality. *Abl^2^* did not suppress the lethality, but all four *fax* alleles, *fax^M7^*, *fax^KG05016^*, *fax^EY01882^*, and *fax^BG00833^*, suppressed this induced lethality (Table 1, Rows 72−76).

Five deficiencies that span the **78D5;80C2 region** were found to be suppressors. In this region *mushroom-body expressed* (*mub^04093^*) (Grams and Korge 1998) did not suppress the *OK107 > unf*-induced lethality, but *Tenascin major* (*Ten-m^05309^*) (Hong *et al.* 2012; Mosca *et al.* 2012; Zheng *et al.* 2011) did suppress the lethality (Table 1, Rows 91, 92). In the **78D5;80C2 region**, three deficiencies suppressed the lethality. The two candidates, *NMDA Receptor 1* (*NMDAR1*) (Xia *et al.* 2005) and *Ras homolog enriched in brain ortholog* (*Rheb*) (Brown *et al.* 2012; Yaniv *et al.* 2012), are found in all three of these deficiencies. However, only *Rheb^EY08085^* suppressed the *OK107 > unf*-induced lethality (Table 1, Rows 98, 99). *Df(3R)BSC507* is a small deficiency in which *Fps oncogene analog* (*Fps85D*; also known as *Fer*) (Murray *et al.* 2006) was the only candidate gene identified. The *Fps85D^X21^* allele suppressed the *OK107 > unf*-induced lethality (Table 1, Row 101). In the **89A8;89B2 region**, two deficiencies and the *Synapse-associated protein 47kD* (*Sap47^EY07944^*) (Reichmuth *et al.* 1995; Saumweber *et al.* 2011) allele suppressed the *OK107 > unf*-induced lethality (Table 1, Row 110). Three deficiencies in the **89B6;89B18 region** suppressed the lethality. *gilgamesh* (*gish*) is a likely candidate based on its previously described expression and function in the MBs (Tan *et al.* 2010) and the fact that it is found in all three of these deficiencies. The *gish^KG03891^* allele suppressed the *OK107 > unf*-induced lethality (Table 1, Row 116). The **95E1;96A13 region** includes five overlapping deficiencies that were identified as suppressors. Five candidate genes that were found in one or more of these deficiencies include *Tsc1* (*Tsc1*) (Yaniv *et al.* 2012), *Syntaxin 1a* (*Syx1a*) (Lagow *et al.* 2007; Wu *et al.* 1999), *jaguar* (*jar*) (Kisiel *et al.* 2011), *Syntaxin 18* (*Syx18*) (Littleton 2000), and *slowpoke* (*slo*) (Atkinson *et al.* 2000; Lee and Wu 2010). Of these five candidate genes, the *Tsc1^F01910^*, *jar^1^*, and *slo^1^* alleles suppressed the *OK107 > unf*-induced lethality (Table 1, Rows 128−132). Although likely candidates were not identified for the **98B6;99A1 region** defined by three overlapping deficiencies, *Dopamine 1-like Receptor 2* (*DopR2*; also known as *DAMB*), a gene with well-established roles in MB-associated behaviors (Berry *et al.* 2012; Chen *et al.* 2012; Draper *et al.* 2007; Selcho *et al.* 2009; Seugnet *et al.* 2008) was accidentally selected as a candidate gene and tested due to a misunderstanding of the limits of one of these original deficiencies. This error was noted only after *DopR2* had been thoroughly tested. Small-eyed flies were not observed when the *DopR2^MB05107^* allele was tested (Table 1, Row 138). In the adjacent **99D1;99D8 region**, two overlapping deficiencies and *axin* (*axn^EY10228^*) (Chiang *et al.* 2009; Hida *et al.* 2012), the only allele tested, suppressed the lethality (Table 1, Row 143). Lastly, four deficiencies spanning the **100A;100E3 region** were identified as suppressors. Of the four candidate genes that were tested, two hypomorphic alleles of *tailless* (*tll*) (Kurusu *et al.* 2009), *tll^1^* and *tll^149^*, and *discs overgrown* (*dco^3^*) (Yamazaki *et al.* 2007) suppressed the *OK107 > unf*-induced lethality. *tramtrak* (*ttk^le11^*) (Nicolai *et al.* 2003) did not (Table 1, Rows 151−154).

### Phenotypic analysis of MBs in animals doubly heterozygous for *unf* and single candidate genes

To test whether *unf*-interacting genes identified in the suppressor screen impact MB development in an *unf*-dependent manner, mutant alleles of candidate genes that suppressed the *OK107 > unf*-induced lethality were crossed to *;unf^X1^UASmCD8/CyO;;OK107/+* or *;unf^X1^/CyO* mutants to generate animals that were heterozygous for both *unf* and a specific candidate gene. The experimental rationale is based on the idea that if a candidate gene acts downstream of *unf* and is required for the development of any or all of the five MB lobes, then reducing the dosage of *unf* and such a downstream gene may compromise the developmental process resulting in one or more defective lobes. To test this hypothesis and determine whether any of these candidate genes play a role in MB development, brains of progeny heterozygous for a candidate gene and heterozygous for the *unf^X1^* mutant allele were processed immunohistochemically and the MB morphologies were analyzed by confocal microscopy. Of the 19 candidate genes, *axn*, *Fps85D*, *Tsc1*, *Rheb*, *msn*, and *Sytβ* significantly impacted MB development (Table 2 and Figure 2). *DopR2* was mistakenly tested in doubly heterozygous animals and also significantly impacted MB development (Table 2 and Figure 2).

**Table 2:** Genetic interactions between *unf* and candidate genes

Row	Genotype	MB Defects				
		Missing Medial Axons (%)	Missing Dorsal Axons (%)	Misproject-ions (%)	Midline Crossing (%)	n
Controls						
1	*w^1118^*	0	0	0	0	10
2	*;unf^X1^/+*	0	0	0	0	15
3	*;UASmCD8/+;;OK107/+*	0	6	0	0	18
4	*;unf^X1^UASmCD8/+;;OK107/+*	0	0	0	8	12
5	*;UASmCD8/+;axn^EY10228^/+;OK107/+*	30	0	0	0	10
6	*;;axn^EY10228^/+*	11	0	0	0	18
7	*;UASmCD8/+;Fps85D^X21^/+;OK107/+*	0	0	0	0	14
8	*;;Fps85D^X21^/+*	0	0	0	0	14
9	*;UASmCD8/+;Rheb^EY08085^/+;OK107/+*	0	0	0	0	10
10	*;UASmCD8/+;Tsc1^F01910^/+;OK107/+*	0	0	0	0	8
11	*;UASmCD8/+;msn^102^/+;OK107/+*	0	0	0	0	13
12	*;UASmCD8/+;DopR2^MB05107^/+;OK107/+*	0	0	0	0	14
13	*;UASmCD8/+;fax^M7^/+;OK107/+*	0	0	0	0	12
14	*;UASmCD8/+;fax^BG00833^/+;OK107/+*	0	0	0	0	8
15	*;UASmCD8/+;fax^KG05016^/+;OK107/+*	0	0	0	0	7
16	*;;fax^M7^/+*	0	0	0	7	15
17	*;UASmCD8/+;Sytβ^PL00192^/+;OK107/+*	7	0	0	7	15
18	*;UASmCD8/+;Sytβ^BG02150^/+;OK107/+*	13	0	0	0	8
19	*;UASmCD8/+;dlt^04276^αSpec^04276^/+*; *OK107/+*	0	0	0	0	13
20	*;UASmCD8/+;tll^1^/+;OK107/+*	0	0	0	0	6
21	*;UASmCD8/+;tll^149^/+;OK107/+*	0	0	0	0	7
22	*;UASmCD8/+;slo^1^/+;OK107/+*	0	0	0	0	10
Double heterozygotes						
23	*;unf^X1^UASmCD8/+;axn^EY10228^/+;OK107/+*	77**^,^[*]	0	0	0	13
24	*;unf^X1^/+;axn^EY10228^/+*	41*^,^[*]	0	0	0	17
25	*;unf^X1^UASmCD8/+;Fps85D^X21^/+;OK107/+*	40**^,^[*]	0	0	0	10
26	*;unf^X1^/+;Fps85D^X21^/+*	30*^,^[*]	5	0	0	20
27	*;unf^X1^UASmCD8/+;Rheb^EY08085^/+;OK107/+*	27*^,^[*]	0	0	0	11
28	*;unf^X1^UASmCD8/+;msn^102^/+;OK107/+*	27*^,^[*]	7	0	40	15
29	*;unf^X1^UASmCD8/+;Tsc1^F01910^/+;OK107/+*	20[*]	0	0	0	10
30	*;unf^X1^UASmCD8/+;DopR2^MB05107^/+;OK107/+*	6	31*^,^[*]	0	0	16
31	*;unf^X1^UASmCD8/+;fax^M7^/+;OK107/+*	0	15	8	8	13
32	*;unf^X1^UASmCD8/+;fax^BG00833^/+;OK107/+*	0	14	0	0	7
33	*;unf^X1^UASmCD8/+;fax^KG05016^/+;OK107/+*	0	9	0	0	11
34	*;unf^X1^UASmCD8/+;fax^EY01882^/+;OK107/+*	0	0	0	0	10
35	*;;fax^M7/M7^*	0	0	25[*]	8	12
36	*;unf^X1^UAS-mCD8/+;Sytβ^PL00192^/+;OK107/+*	10	0	30*^,^[*]	10	10
37	*;unf^X1^UAS-mCD8/+;Sytβ^BG02150^/+;OK107/+*	20	10	20	20	10
38	*;unf^X1^UASmCD8/+;dlt^04276^αSpec^04276^/+;OK107/+*	14	14	0	0	14
39	*;unf^X1^UASmCD8/+;tll^1^/+;OK107/+*	17	17	0	17	6
40	*;unf^X1^UASmCD8/+;tll^149^/+;OK107/+*	0	17	0	33	6
41	*;unf^X1^UASmCD8/+;slo^1^/+;OK107/+*	5	5	5	10	19
42	*;unf^X1^UASmCD8/+;αSpec^lm88^/+;OK107/+*	0	0	0	0	10
43	*;unf^X1^UASmCD8/+;Shab^MB027261^/+;OK107/+*	0	0	0	0	7
44	*;unf^X1^UASmCD8/+;aay^S042314^/+;OK107/+*	0	0	0	0	10
45	*;unf^X1^UASmCD8/+;αTub67C^1^/+;OK107/+*	0	0	0	0	10
46	*;unf^X1^UASmCD8/+;caps^02937^/+;OK107/+*	0	0	0	0	9
47	*;unf^X1^UASmCD8/+;mub^04093^/+;OK107/+*	0	0	0	0	8
48	*;unf^X1^UASmCD8/+;Ten-m^05309^/+;OK107/+*	0	0	0	0	10
49	*;unf^X1^UASmCD8/+;Sap47^EY07944^/+;OK107/+*	0	0	0	0	11
50	*;unf^X1^UASmCD8/+;gish^KG03891^/+;OK107/+*	0	0	0	0	10
51	*;unf^X1^UASmCD8/+;jar^1^/+;OK107/+*	0	0	0	0	11
52	*;unf^X1^UASmCD8/+;dco^j3B9^;OK107/+*	0	0	0	0	10
53	*;unf^X1^UASmCD8/+;ttk^1e11^;OK107/+*	0	0	0	0	7

Data are presented as percentages of whole brains that exhibit the phenotype. Asterisks indicate one-tailed *p*-values of <0.05 from Fisher’s exact test. *unf*, unfulfilled; MB, mushroom body. Midline crossing defects were not included in the statistical analyses. Although mub, ttk, and DopR2, were not suppressors of the OK107. unf-induced lethality, these genes were included in the secondary phenotypic screen based on their expression in the MB. UASmCD8 = UASmCD8::GFP, OK107 = OK107-GAL4.

** The rate at which MB defects were observed in double heterozygotes differed significantly from the rate at which they were observed in each of the appropriate individual control groups when tested in pair-wise combinations

* The rate at which MB defects were observed in double heterozygotes differed significantly from the rate at which they were observed in at least one of the appropriate individual control groups.

^[^*^]^The rate at which MB defects were observed in double heterozygotes differed significantly from the rate at which they were observed when tested in a single pair-wise combination with the appropriately pooled controls.

**Figure 3:**
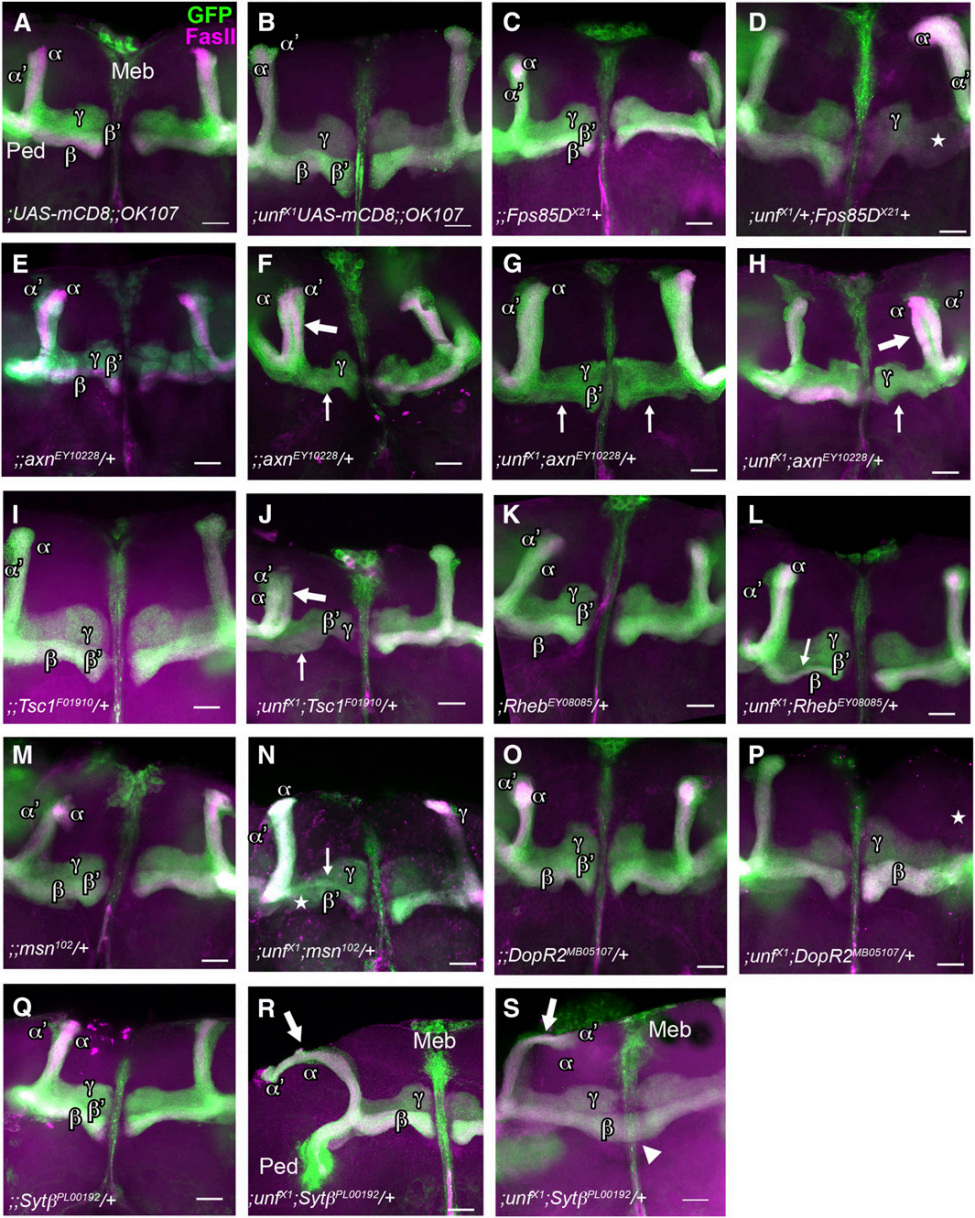
Mushroom body (MB) phenotypes in animals doubly heterozygous for *unfulfilled* (*unf*) and single candidate genes. In the adult brain, the MB is a paired neuropil structure composed of three subtypes of MB neurons, γ, α´/β´, and α/β. Each neuron projects dendrites that contribute to a large dendritic field (calyx) and an axon that travels anteroventrally. MB axons fasciculate with other MB axons, forming a peduncle (Ped) before branching and projecting axons medially and dorsally. α´ and α axons project dorsally, whereas the adult γ and the β´ and β axons project medially, forming five distinctive lobes. To visualize the MB lobes, *OK107-GAL4* (*OK107*) was used to drive expression of the *UAS-mCD8*::*GFP* (*UASmCD8*) transgene in all MB neurons and their axons (green). Lobes were distinguished by using anti-Fas II to label α and β lobes (magenta). Note that the *OK107* and *UASmCD8* transgenes that are present in all control and experimental animals were not included in the genotypes (C−S) due to limited space in the figure. (A, B) In *;UAS-mCD8;;OK107* and *;unf^X1^UAS-mCD8;;OK107* control animals, all five MB lobes have formed in each of the two brain hemispheres. (C) In *;UAS-mCD8/+;Fps85D^X21^/+;OK107/+* heterozygote controls, all MB lobes are present. (D) In this *;unf^X1^UAS-mCD8/+;Fps85D^X21^/+;OK107/+* double heterozygote, both β´ and β (medial) lobes are missing in the right hemisphere (star). (E, F) *;UAS-mCD8/+;axn^EY10228^/+;OK107* heterozygotes either exhibit the wild type phenotype in which all MB lobes are present, or a mutant phenotype in which β lobes are missing (thin arrow in F). In this case the missing β lobe appears to have misprojected dorsally (thick arrow in F). (G, H) In *;unf^X1^UAS-mCD8/+;axn^EY10228^/+;OK107* double heterozygotes, β lobes are missing in one or both brain hemispheres (thin arrows in G and H) or β lobes have misprojected dorsally alongside the α (dorsal; magenta) lobe (thick arrow in H). (I) All MB lobes are present in *;UAS-mCD8/+;Tsc1^F01910^/+;OK107* heterozygote controls. (J) In this *;unf^X1^UAS-mCD8/+;Tsc1^F01910^/+;OK107* double heterozygote, the missing β lobe (thin arrow) appears to have misprojected dorsally (thick arrow) in the left brain hemisphere. (K) In *;UAS-mCD8/+;Rheb^08085^/+;OK107* heterozygotes, all MB lobes have formed. (L) In this *;unf^X1^UAS-mCD8/+;Rheb^08085^/+;OK107* double heterozygote, the β (medial; magenta) lobe appears thin in the left hemisphere (thin arrow). (M) In this *;UAS-mCD8/+;msn^102^/+;OK107* heterozygote, all MB lobes have formed. (N) In this *;unf^X1^UAS-mCD8/+;msn^102^/+;OK107* double heterozygote, the β´ lobe is thin (thin arrow), and the β lobe is missing (star). (O) In *;UAS-mCD8/+;DopR2^MB05107^/+;OK107* heterozygotes, all MB lobes have formed. (P) In this *;unf^X1^UAS-mCD8/+;DopR2^MB05107^/+;OK107* double heterozygote, both α´ and α (dorsal) lobes are missing (star) in the right brain hemisphere. (Q) In this *;UAS-mCD8/+;Sytβ^PL00192^/+;OK107* heterozygote, all MB lobes have formed. (R, S) In *;unf^X1^UAS-mCD8/+;Sytβ^PL00192^/+;OK107* double heterozygotes, both α´ and α (dorsal) lobes misproject making sharp bends in either direction where they normally should have stopped growing (thick arrow in R and S). Note that medial axons cross the midline in S (arrowhead). Anterior is always up and the midline is in the center with the exception of R and S. Due to the nature of the defect in R and S, only the left brain hemisphere is completely visible. Ped, peduncle; Meb, median bundle. Scale bars = 25 μm.

**Figure 4:**
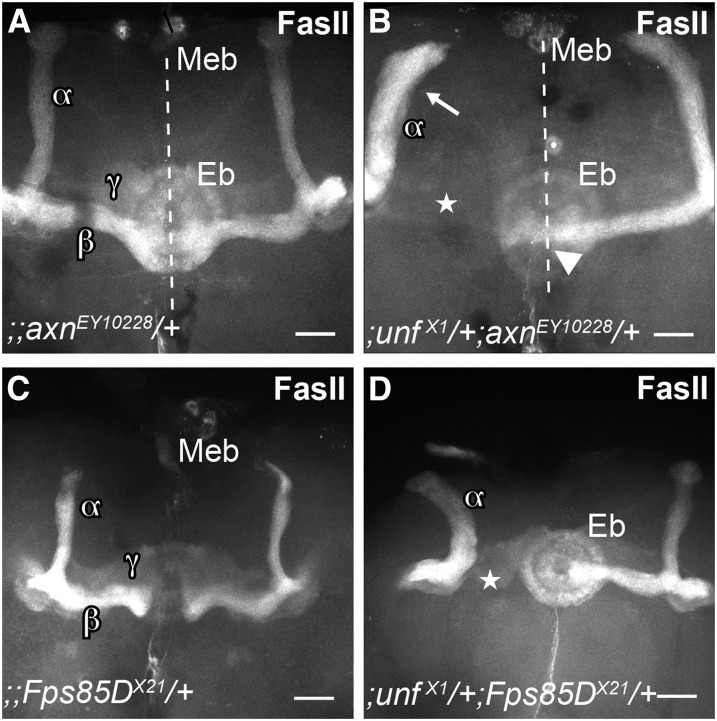
Double heterozygotes without the *UAS-mCD8GFP* and *OK107-GAL4* transgenes exhibit the same mushroom body (MB) phenotypes as those containing these transgenes. Adult brains of experimental and control animals were labeled with anti-Fas II to visualize only α/β projections. (A) All labeled MB lobes are present in this *;;axn^EY10228^/+* heterozygote. (B) In the left hemisphere of this *;unf^X1^;axn^EY10228^/+* double heterozygote, the β (medial) lobe is missing (star) and the α (dorsal) lobe appears thick (arrow) suggesting that the β axons have misprojected dorsally. In the right hemisphere, the α and β lobes are present, but the β lobe crosses the midline (dotted line) (arrowhead). (C) All labeled MB lobes are present in this *;;Fps85D^X21^/+* heterozygote. (D) In this *;unf^X1^;Fps85D^X21^/+* double heterozygote, the β lobe is missing (star) in the left hemisphere. Eb, ellipsoid body; Meb, median bundle. Scale bars = 25 μm.

Five double heterozygotes were primarily missing β′ and/or β (medial) axons. *axin*, *Tsc1*, and *Rheb* impacted primarily the β lobe, whereas *Fps85D* and *msn* impacted both β′ and β lobes. Although not fully penetrant, *;unf^X1^/+;axn^EY10228^/+* double heterozygotes were missing medial β lobes in one or both hemispheres at frequencies that were significantly different than each of the individual control groups (Table 2, Rows 23, 3, 4, 5; and Figure 3, H and I). In at least one animal, α/β axons branched at the end of the peduncle and instead of the β lobe projecting medially, the β lobe projected dorsally with the α lobe, suggesting that *axn* plays a role in the guidance of β axon branches (Figure 3H). Because MB defects were occasionally observed in *;UASmCD8/+;;OK107/+* controls, it is possible that MB defects could be due to the insertion of either transgene and/or the presence of the GFP or GAL4 proteins. To address this possibility, double heterozygotes and heterozygote controls without the *OK107-GAL4* and *UAS-mCD8* transgenes were labeled with anti-Fas II and analyzed. In these *;unf^X1^/+;axn^EY10228^/+* double heterozygotes β lobes were missing but at lower frequency than animals containing *OK107-GAL4* and *UAS-mCD8* (Table 2, Row 24; and Figure 4B). These data suggest that the presence of the transgenes potentiates the missing β-lobe phenotype observed in *;unf^X1^/+;axn^EY10228^/+* double heterozygotes. However, the frequency at which β lobes were missing in *;unf^X1^/+;axn^EY10228^/+* animals without the transgenes was significantly different than the appropriately pooled controls of the same genetic background (Table 2, Rows 24, 2, 6).

Similarly, in *;unf^X1^/+;Tsc1^F01910^/+* (*;unf^X1^UAS-mCD8/+*; *Tsc1^F01910^/+;OK107/+*) double heterozygotes β lobes were missing or misguided dorsally at frequencies that differed significantly from the appropriately pooled controls (Table 2, Rows 29, 3, 4, 10; Figure 3J). In *;unf^X1^*/+*;Rheb^EY08085^/+* (*;unf^X1^UAS-mCD8/+;Rheb^EY08085^/+;OK107/+*) animals β lobes were thin, suggesting that at least some of the medial axons stalled or misprojected dorsally (Figure 3L). The rate at which these defects were observed differed significantly from controls (Table 2, Rows 27, 3, 4, 9).

Both β′ and β lobes were missing in *;unf^X1^/+;Fps85D^X21^/+* (*;unf^X1^UAS-mCD8/+;Fps85D^X21^/+;OK107/+*) animals at frequencies that were significantly different than each of the individual control groups (Table 2, Rows 25, 3, 4, 7; and Figure 3D). In these animals these medial axons appeared to stall prior to axon branching. In addition, double heterozygotes without the *OK107-GAL4* and *UAS-mCD8* transgenes exhibited the same phenotype at frequencies that were significantly different than controls of the same genetic background (Table 2, Rows 26, 2, 8; and Figure 4D). The frequency of aberrant phenotypes of *;unf^X1^/+;Fps85D^X21^/+* double heterozygotes without the transgenes was slightly lower than that of experimental animals with the transgenes (Table 2, Rows 25, 26). Thus, like the *unf:axn* interaction, the *unf:Fps85D* interaction is sensitive to the presence of the *OK107-GAL4* and *UAS-mCD8* transgenes.

Both β′ and β (medial) lobes were thin or missing in *;unf^X1^/+;msn^102^/+* (*;unf^X1^UAS-mCD8/+;msn^102^/+;OK107/+*) animals at frequencies that were significantly different from controls (Table 2, Rows 28, 3, 4, 11; and Figure 3N). In these animals medial axons sometimes appeared disorganized and crossed the midline. However, since midline crossing defects are highly sensitive to genetic and environmental backgrounds (Chang *et al.* 2008; Michel *et al.* 2004), midline crossing defects were omitted from our analyses.

**Figure 5:**
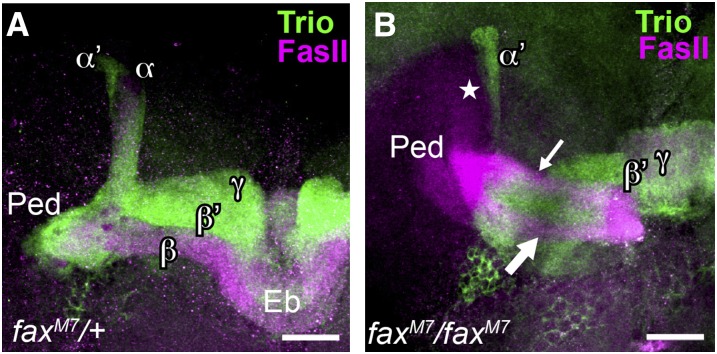
*fax* homozygotes exhibit α (dorsal) axon misprojections. Brains of experimental and control animals were double-labeled with anti-Fas II to visualize α/β neurons, and anti-Trio to visualize γ and α´/β´ neurons. (A) In this *;;fax^M7^/+* heterozygote all five mushroom body lobes are present. (B) In this *;;fax^M7/M7^* homozygote, the α (dorsal) lobe is missing (star) and two distinct Fas II-positive axon bundles project medially (arrow) alongside the γ and β´ (medial) lobes. The presence of the two Fas II-positive medially projecting bundles suggests that one is the β lobe (thick arrow) and the other is the misprojected α lobe (thin arrow). Ped, peduncle; Eb, ellipsoid body. Scale bars = 10 μm.

Defects in α′ and α (dorsal) lobes were observed primarily in double heterozygotes containing *unf* and *DopR2* or *Sytβ*, and in *fax* homozygotes. In *;unf^X1^/+;DopR2^MB05107^/+* (*;unf^X1^UASmCD8/+;DopR2^MB05107^/+;OK107/+*) double heterozygotes, both α′ and α lobes were missing at frequencies that were significantly different from controls (Table 2, Rows 30, 3, 4, 12; and Figure 3P). This result was unexpected because *DopR2* did not suppress the *OK107 > unf*-induced lethality. Although α lobes were missing in double heterozygotes for three of four different *fax* alleles (*;unf^X1^UASmCD8/+;fax^M7^/+;OK107/+*, *;unf^X1^UASmCD8/+*; *fax^BG00833^/+;OK107/+*, and *;unf^X1^UASmCD8/+*; *fax^KG05016^/+;OK107/+*) the rate of occurrence did not differ significantly from any single control group or pooled controls (Table 2, Rows 31, 32, 33, 3, 4, 13, 14, 15). Prior to thorough statistical analysis and because *fax^M7^* mutants are homozygous viable, we examined the MBs in *;;fax^M7/M7^* homozygotes. Interestingly, in these animals, we observed that α lobes misprojected medially alongside the β lobe (Figure 5B). These defects were observed at frequencies that were significantly different than the appropriately pooled controls (Table 2, Rows 35, 1, 2, 16). These data suggest that *fax* may play a role in the guidance of branches that form the α lobe but that the role of *fax* in this context is independent of *unf*.

*;unf^X1^/+;Sytβ^PL00192^/+ (;unf^X1^UASmCD8/+;GAL4D,EYFP,Sytβ^PL00192^/+;OK107/+)* and *;unf^X1^/+;Sytβ^BG02150^/+ (;unf^X1^UASmCD8/+;Sytβ^BG02150^/+;OK107/+*) double heterozygotes shared a unique dorsal axon phenotype in which α′ and α axons misprojected making sharp turns or bends where they normally should have stopped growing (Figure 3, R and S). The frequency at which dorsal misprojections were observed in *;unf^X1^/+;Sytβ^PL00192^/+* animals differed significantly from controls (Table 2, Rows 36, 3, 4, 17). The fact that the *Sytβ^PL00192^* allele, but not the *Sytβ^BG02150^* allele, significantly impacted MB development is consistent with the *Sytβ^PL00192^* allele-specific suppression of the *OK107* > unf-induced lethality and the suggestion that the *Sytβ^PL00192^* is an amorph or a more severe hypomorph than the *Sytβ^BG02150^* allele (Table 1, Row 61). Additional MB defects including the absence of medial or dorsal lobes or stubby dorsal lobes were occasionally observed in experimental and control animals containing the *Sytβ^PL00192^* or *Sytβ^BG02150^* alleles, suggesting that *Sytβ* alleles may cause some interesting MB phenotypes independent of *unf*, but the dorsal misprojection phenotype was never observed in any controls demonstrating that *Sytβ* regulates dorsal axon growth and guidance in an *unf*-dependent manner.

## Discussion

This genetic suppressor screen followed by a secondary phenotypic screen resulted in the identification of seven genes (*axn*, *Tsc1*, *Rheb*, *Fps85D*, *msn*, *DopR2*, and *Sytβ*) that impact MB neuron development in an *unf*-dependent manner. *Rheb* and *DopR2* are known to be expressed in the MB and validate our screen. *axn*, *Fps85D*, *msn*, and *Sytβ* were previously unknown to be involved in MB development.

Five genes impacted primarily medial MB lobes. Animals doubly heterozygous for *unf* and *axn*, *Tsc1*, *Rheb*, *Fps85D*, or *msn* exhibited similar MB defects in which β′ and/or β medial lobes were not observed, were thin, or misprojected dorsally. Dorsal lobes were normal in these animals, suggesting branch-specific roles for these genes. In some *;unf/+;axn/+* double heterozygotes, medial axons clearly misprojected. Occasionally, thick dorsal lobes or two distinct Fas II-positive dorsal lobes were observed in these animals suggesting that *axn* is required for the proper guidance of the β branch of the α/β neuron (Figure 3H). However, it is difficult to know whether β axons always misproject or if they sometimes stall, and if stalling occurs prior to or after branching.

Our interpretation of the *;unf/+;Fps85D/+* phenotype in which β′ and β axons appeared to spread out and stall at the choicepoint and that two Fas II-positive dorsal projections were never observed in these animals suggests that *Fps85D* may play a role in medial axon growth and branching, whereas *axn* may only be required for the later guidance of β axon projections. We are now generating *axn* and *Fps85D* mutant MARCM clones to understand better the nature of these medial MB axon defects.

Axn, Fps85D, Tsc1, and Rheb are components of intracellular signaling cascades that may converge to regulate the necessary cellular changes required for medial MB lobe development. Each of these are directly or indirectly associated with the Wingless/Wnt pathways. Both the canonical and noncanonical Wnt pathways have been implicated in many biological processes including neuronal development. In canonical Wnt signaling, transduction through the Frizzled (Fr) receptor facilitates β-catenin relocalization to the nucleus, where it functions as a transcriptional co-activator. In the absence of Wnt signaling, the GSK3β/APC/Axn (glycogen synthase kinase-3β/adenomatous polyposis coli/axin) complex phosphorylates β-catenin targeting it for degradation (Clevers and Nusse 2012; Putzke and Rothman 2010; Salinas and Zou 2008). In the noncanonical context, β-catenin functions as a component of membrane adhesion complexes. Components of the Wnt noncanonical pathway activate additional intracellular signaling cascades that directly regulate cytoskeletal reorganization (Lai *et al.* 2009). In *Drosophila*, WNT family proteins regulate MB axon differentiation via cell-surface receptors and planar cell polarity protein interactions activating the Wnt noncanonical pathway (Grillenzoni *et al.* 2007; Ng 2012; Shimizu *et al.* 2011; Soldano *et al.* 2013). In particular, loss-of-function mutants of the Wnt/planar cell polarity pathway show a range of MB branching defects. Removing different components alters the bias toward the production of medial or dorsal branches (Ng 2012). In contrast, we show that AXN, a component of the canonical Wnt pathway, is required for the normal patterning of MB β medial branches specifically. It is possible that AXN regulates the growth or guidance of medial axons by regulating levels of β-catenin and as a result β-catenin-mediated activation of target genes. Additional support for the involvement of the canonical Wnt pathway in MB medial lobe development is that *shaggy* (*sgg*)/GSK3β has been identified as a potential target of *unf* via RNA transcriptome analysis (J. Molnar, unpublished data). *sgg*/GSK3β could not have been identified in our third chromosome suppressor screen because it is on the X chromosome. Interestingly, a recent study showed that the GSK3β/Axin-1/β-catenin complex regulates responsiveness to the repulsive cue Semaphorin3A (Sema3A) via regulation of endocytic processes in chick dorsal root ganglion neurons, providing a model by which Axn regulates axon guidance independent of gene transcription (Hida *et al.* 2012). Furthermore, interactions between downstream Wnt component Disheveled (Dvl) and Axn have been shown to regulate MTs in the cytoskeleton directly *in vitro* (Ciani *et al.* 2004).

In MBs, AXN and FPS85D may act together to regulate the development of medial MB lobes. *Fps85D* encodes a nonreceptor protein tyrosine kinase that functions in many morphological processes via the regulation of adhesion mechanisms and reorganization of the MT and actin cytoskeleton (reviewed by Greer 2002). In *Drosophila*, FPS85D is expressed at the leading edge of migrating cells, where it cooperates with SRC42A in the phosphorylation of β-catenin at adherens junctions to regulate dorsal closure. *Fps85D* is also expressed in embryonic central nervous system neurons and glia (Murray *et al.* 2006). However, FPS85D-mediated axon guidance has not been demonstrated in flies. Interestingly, FRK-1, the C. *elegans* ortholog of *Drosophila* FPS85D, represses Wnt signaling by sequestering β-catenin in adhesion complexes (Putzke and Rothman 2010). Thus, AXN and FPS85D may regulate medial MB lobe development via regulation of Wnt signaling or via reorganization of the cytoskeleton directly.

Yaniv *et al.* (2012) demonstrated that *unf* regulates MB γ axon re-extension via the Tsc1/Rheb/Tor/S6K pathway (Yaniv *et al.* 2012). We identified *Tsc1* and *Rheb*, but not *S6K*, as suppressors of the *OK107 > unf*-induced lethality, and found that medial lobes were thin, missing, or misprojecting in animals doubly heterozygous for *unf* and *Tsc1* or *Rheb*. The observation of thin medial lobes in *;unf/+;Rheb/+* animals is consistent with a requirement for *Rheb* for γ axon re-extension. The results for *Tsc1* were unexpected because UNF activates the Tor pathway by repressing *Tsc1* in flies (Yaniv *et al.* 2012), and the mouse ortholog of *unf*, *Nr2e3*, negatively regulates *Tsc1* in mice (Haider *et al.* 2009). The fact that γ MB lobes appeared normal and that in the developing visual system *Tsc1* mediates photoreceptor axon guidance and synaptogenesis independent of the Rheb/Tor/S6K pathway suggests that alternative mechanisms are likely to exist (Knox *et al.* 2007).

Animals doubly heterozygous for *unf* and *DopR2* or *Sytβ* exhibited MB defects in which dorsal lobes were missing (*DopR2*) or extended beyond the termination point (*Sytβ*).

*DopR2* and *Sytβ* encode synaptic proteins. Although *DopR2* roles in MB-associated behaviors, including α′ and α lobe-mediated long-term memory formation is well documented, a role for *DopR2* in neuron differentiation has not been demonstrated. One possible mechanism for DOPR2-mediated axon growth and guidance in MB neurons is via activation of intracellular signaling pathways resulting in modulation of axon guidance cues and cytoskeletal proteins. For example, drug-induced activation of dopamine D_1_ receptors resulted in increased cyclic adenosine monophosphate (cAMP) levels and down-regulated *EphB1*, *DCC*, and *Sema3C* gene expression *in vitro* (Jassen *et al.* 2006). Furthermore, asymmetric localization and activation of cAMP and other intracellular molecules suggests an underlying mechanism for neuron branching as well as branch-specific behavior. In *Drosophila*, bath application of dopamine on a fly brain *in vitro* resulted in a uniform increase of cAMP across the MB, but when dopamine was administered to the brain of a living fly, cAMP-dependent protein kinase activity was α lobe-specific, suggesting that intracellular components of dopamine signaling cascades are differentially coupled within axon branches of the same neuron (reviewed by Waddell 2010). SYTβ is likely to influence axon growth and guidance via membrane dynamics. In the fly brain, SYTα is reportedly expressed in large central nervous system neurons as well as the larval MB, whereas SYTβ is expressed in pars intercerebralis neurons (Adolfsen *et al.* 2004). These expression patterns suggest roles for synaptotagmins in both the trafficking and release of neurotransmitters as well as neuropeptides throughout the nervous system (Adolfsen *et al.* 2004). It is possible that SYTβ is expressed in the adult MB and acts autonomously in the dorsal lobes, where it functions in activity-dependent axon growth and guidance. Alternatively, it is possible that SYTβ functions nonautonomously in nearby pars intercerebralis neurons via modulation of neuropeptides that may be required for the termination of α′ and α (dorsal) axons.

Of the 19 genes that suppress the *OK107 > unf*-induced lethality, only six also impacted MB development in our secondary phenotypic screen. The remaining 13 genes do not result in gross morphologic defects of the MB. Some of these 13 may be *unf*-dependent genes involved in eclosion or other processes that contribute to survivability. At least three (*Sap47*, *Shab*, and *slo*) of these 13 genes are associated with synaptic activity and plasticity and may be required for neuronal activity without impacting MB morphology.

**Figure 6:**
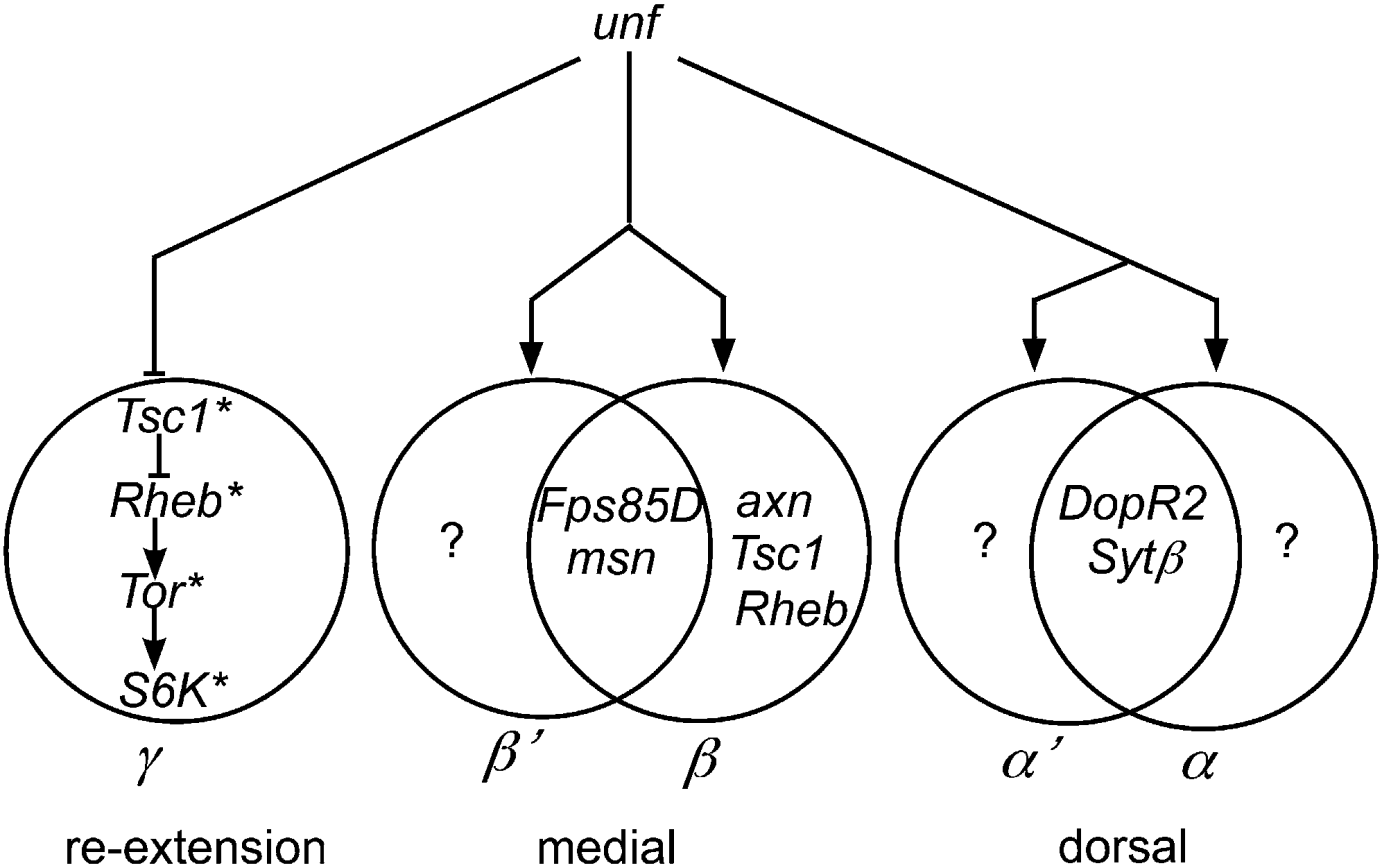
Roles for *unfulfilled* (*unf*)-interacting genes in the formation of adult-specific branches. This schematic shows that unf negatively regulates the Tsc1/Rheb/Tor/S6K pathway required for adult γ re-extension (Yaniv *et al.*, 2012). The data presented here show that *unf*-interacting genes have been identified that are involved in both β´ and β lobe formation, β lobe formation only, and both α´ and α lobe formation. This model predicts that there are other *unf*-interacting genes that specifically control β´ lobe formation, α´ lobe formation, and α lobe formation only.

We have used a series of Venn diagrams to summarize the roles of *unf*-dependent genes that have been identified in this screen or by others (Yaniv *et al.* 2012) (Figure 6). This model suggests that there are additional classes of genes that regulate the development of larval γ branches, β′ branches, and α′ or α branches. The identification of genes involved in the development of larval γ branches is of particular interest because of the possibility that the γ neurons establish the pioneer tracts that are essential for later MB axon pathfinding and branching.

## Supplementary Material

Supporting Information
