# Supplementary material for: unfulfilled Interacting Genes Display Branch-Specific Roles in the Development of Mushroom Body Axons in Drosophila melanogaster
Source: G3 (Bethesda). 2014 Feb 20;4(4):693–706. doi: 10.1534/g3.113.009829 (PMC4577660; doi:10.1534/g3.113.009829)
Supplement: Supporting Information [file supp_4_4_693__index.html]

unfulfilled Interacting Genes Display Branch-Specific Roles in the Development of Mushroom Body Axons in Drosophila melanogaster — Supporting Information 

# *unfulfilled* Interacting Genes Display Branch-Specific Roles in the Development of Mushroom Body Axons in *Drosophila melanogaster*

## Supporting Information for Bates *et al.*, 2014

**Files in this Data Supplement:**

- Supporting Information - File S1 and Table S1 (PDF, 234 KB)
- File S1 - Supporting Methods (PDF, 175 KB)
- Table S1 - Deficiencies that fail to suppress the lethality induced by ectopic expression of *unfulfilled*. (PDF, 157 KB)
