# Supplementary material for: unfulfilled Interacting Genes Display Branch-Specific Roles in the Development of Mushroom Body Axons in Drosophila melanogaster
Source: G3 (Bethesda). 2014 Feb 20;4(4):693–706. doi: 10.1534/g3.113.009829 (PMC4577660; doi:10.1534/g3.113.009829)
Supplement: Supporting Information [file supp_g3.113.009829_009829SI.pdf]

G3 · Genes | Genomes | Genetics

unfulfilled interacting genes display branch-specific roles in the development of mushroom body axons in *Drosophila melanogaster*

Karen E. Bates, Carl Sung, Liam Hilson, and Steven Robinow
Department of Biology, University of Hawaii, Honolulu, HI 96822

Corresponding author:
Steven Robinow
University of Hawaii, Department of Biology
2538 McCarthy Mall, Edmondson Hall
Honolulu, Hawaii 96822
Phone: 808-956-8088
Fax: 808-956-4745
Email: robinow@hawaii.edu

DOI: 10.1534/g3.113.009829

File S1

SUPPORTING METHODS

Candidate genes that were tested that are required for axon pathfinding and targeting include *α-Spectrin (α-Spec*^{lm88}) (GARBE and BASHAW 2007), *misshapen* (*msn*^{102}) (RUAN *et al.* 1999; SU *et al.* 2000), *spätzal* (*spz5*^{E03444}) (ZHU *et al.* 2008), *Kinesin-like protein at 64D* (*Klp64D*^{K1}) (BAQRI *et al.* 2006; RAY *et al.* 1999; SADANANDA *et al.* 2012), *veloren* (*velo*^{EY10127}) (BERDNIK *et al.* 2012), *sugarless* (*sgl*^{08310}) (CHO *et al.* 2012), *Actin-related protein 3* (*Arp3*^{EP3640}) (GONCALVES-PIMENTEL *et al.* 2011), *astray* (*aay*^{S042314}) (SALZBERG *et al.* 1997), *RasGAP1* (*GAP1*^{B2}) (YANG and TERMAN 2012), *α-Tubulin67C* (*α-Tub67C*^{1}) (WANG *et al.* 2007), *tartan* (*trn*^{S064117}) (KURUSU *et al.* 2008), *capricious* (*caps*^{02937}) (ABRELL and JACKLE 2001), *commissureless* (*comm*^{M100380}) (TEAR *et al.* 1996), *failed axon connections* (*fax*^{M7}, *fax*^{BG00833}, *fax*^{EY10882}, *fax*^{KG05016}), *Abl tyrosine kinase* (*Abl*^{2}) (HILL *et al.* 1995; LIEBL *et al.* 2000), *schizo* (*siz*^{EY09677}) (HUMMEL *et al.* 1999), *chromosome bows* (*chb*^{4}) (LEE *et al.* 2004), *Tenascin major* (*Ten-m*^{05309}) (HONG *et al.* 2012; MOSCA *et al.* 2012; ZHENG *et al.* 2011), *Fps oncogene analog* (*Fps85D*^{X21}; also known as *Fer*) (MURRAY *et al.* 2006), *Tropomyosin 1* (*Tm1*^{02299}) (STEPHAN *et al.* 2012), *Specifically Rac1-associated protein 1* (*Sra1*^{EY06562}) (BOGDAN *et al.* 2004), *hedgehog* (*hh*^{2}) (HUANG and KUNES 1996; SALECKER *et al.* 1998), and *axin* (*axn*^{EY10228}) (CHIANG *et al.* 2009; HIDA *et al.* 2012). *discs lost* (*dlt*^{04276}; also known as *DPATJ*), which shares a first untranslated exon with *α-Spec*, regulates photoreceptor morphogenesis and maintenance (NAM and CHOI 2006; PIELAGE *et al.* 2003). Candidate genes that were already known to be involved in MB development include *Ptpmeg* (*Ptpmeg*^{1}) (WHITED *et al.* 2007), *Ras homolog enriched in brain ortholog* (*Rheb*^{EY08085}) (BROWN *et al.* 2012; YANIV *et al.* 2012), *Tsc1* (*Tsc1*^{F01910}), *RPS6-p70-protein kinase* (*S6K*$^{l-1}$) (YANIV *et al.* 2012), *tailless* (*tll*^{1}, *tll*^{149}) (KURUSU *et al.* 2009), and *tramtrak* (*ttk*^{1e11}) (NICOLAI *et al.* 2003). Another group of candidate genes are those involved in synaptic activity, learning and memory, or other MB-associated behaviors. These included *Shaker cognate b (Shab*^{MB02726}) (GASQUE *et al.* 2005), *gryzun* (*gry*^{EY03013}) (AKALAL *et al.* 2011; DUBNAU *et al.* 2003), *dikar* (*dikar*^{d02315}) (AKALAL *et al.* 2011), *Synaptotagminβ* (*Sytβ*^{PL00191}, *Sytβ*^{BG02150}) (MACKLER and REIST 2001), *mushroom-body expressed* (*mub*^{04093}) (GRAMS and KORGE 1998), *NMDA Receptor 1* (*NMDAR1*^{05616}) (XIA *et al.* 2005), *Synapse-associated protein 47kD* (*Sap47*^{EY07944}) (REICHMUTH *et al.* 1995; SAUMWEBER *et al.* 2011), *gilgamesh* (*gish*^{KG03891}) (TAN *et al.* 2010), *daughters against dpp* (*dad*^{J1E4}) (RODAL *et al.* 2011), *Syntaxin 1a* (*Syx1a*$^{\Delta 229}$) (LAGOW *et al.* 2007; WU *et al.* 1999), *jaguar* (*jar*^{1}) (KISIEL *et al.* 2011), *Syntaxin 18* (*Syx18*^{EY08095}) (LITTLETON 2000), *slowpoke* (*slo*^{1}) (ATKINSON *et al.* 2000; LEE and WU 2010), *Dopamine 1-like Receptor 2* (*DopR2*^{MB05108}; also known as *DAMB*) (BERRY *et al.* 2012; CHEN *et al.* 2012; DRAPER *et al.* 2007; SELCHO *et al.* 2009; SEUGNET *et al.* 2008), and *discs overgrown* (*dco*^{3}) (YAMAZAKI *et al.* 2007).

Table S1 Deficiencies that fail to suppress the lethality induced by ectopic expression of *unfulfilled*

Deficiency	Start breakpoints	End breakpoints	Small eye flies (*n*)
Df(3L)ED50002	61A1	61B1	0 (31)
Df(3L)ED4177	61B1	61E2	0 (45)
Df(3L)BSC289	61F6	62A9	0 (46)
Df(3L)ED4191	61C3	62A2	0 (24)
Df(3L)BSC23	62E8	63B5-6	0 (78)
Df(3L)BSC671	63A2	63B11	0 (90)
Df(3L)BSC368	63F1	64A4	0 (91)
Df(3L)ED210	64B9	64C13	0 (167)
Df(3L)BSC884	64D6	64E7	0 (22)
Df(3L)BSC27	64D4-5	64E4-6	0 (134)
Df(3L)Exel8104	65F7	66A4	0 (37)
Df(3L)BSC375	66A3	66A19	0 (23)
Df(3L)BSC815	66C3	66D4	0 (39)
Df(3L)BSC389	66C12	66D8	0 (77)
Df(3L)ED4421	66D12	67B3	0 (43)
Df(3L)AC1	67A2	67D11-13	0 (14)
Df(3L)ED4457	67E2	68A7	0 (4)
Df(3L)ED4475	68C13	69B4	0 (72)
Df(3L)ED4486	69C4	69F6	0 (77)
Df(3L)ED4543	70C6	70F4	0 (131)
Df (3L)BSC774	71F1	72D10	0 (39)
Df(3L)ED4685	73D5	74E2	0 (50) [a]
Df(3L)ED4710	74D1	75B11	0 (50) [a]
Df(3L)BSC775	75A2	75E4	0 (50) [a]
Df(3L)BSC220	75F1	76A1	0 (50) [a]
Df(3L)ED229	76A1	76E1	0 (50) [a]
Df(3L)ED4858	76D3	77C1	0 (122)
Df(3L)BSC839	77B4	77C6	0 (86)
Df(3L)BSC797	77C3	78A1	0 (43)
Df(3L)BSC419	78C2	78D8	0 (31)
Df(3R)ED5147	82E7	83A1	0 (52)
Df(3R)ED5156	82F8	83A4	0 (47)
Df(3R)ED5177	83B4	83B6	0 (32)
Df(3R)BSC633	84B2	84C3	0 (91)
Df(3R)BSC466	84E1	85A10	0 (34)
Df(3R)ED5339	85D1	85D11	0 (64)
Df(3R)BSC476	85D16	85D24	0 (14)
Df(3R)BSC621	85F5	85F14	0 (52)
Df(3R)Exel7321	88A9	88B1	0 (79)
Df(3R)ED5664	88D1	88E3	0 (36)
Df(3R)BSC750	88E2	88E5	0 (39)
Df(3R)ED5705	88E12	89A5	0 (35)
Df(3R)BSC515	88F6	89A8	0 (31)
Df(3R)Exel7328	89A12	89B6	0 (23)
Df(3R)ED10642	89B17	89D5	0 (27)
Df(3R)Exel6270	89B18	89D8	0 (41)
Df(3R)BSC790	90B6	90E2	0 (86)
Df(3R)BSC819	93A2	93B8	0 (50)
Df(3R)Exel6272	93A4	93B13	0 (19)
Df(3R)ED10845	93B9	93D4	0 (22)
Df(3R)BSC677	93D1	93F14	0 (35)
Df(3R)ED6085	93F14	94B5	0 (24)
Df(3R)ED6096	94B5	94E7	0 (40)
Df(3R)BSC137	94F1	95A4	0 (35)
Df(3R)BSC489	94F3	95D1	0 (44)

Df(3R)Exel6196	95C12	95D8	0 (89)
Df(3R)ED6187	95D10	96A7	0 (42)
Df(3R)ED6220	96A7	96C3	0 (33)
Df(3R)BSC461	96B15	96D1	0 (89)
Df(3R)Exel6202	96D1	96D1	0 (50) [a]
Df(3R)Exel6203	96E2	96E6	0 (50) [a]
Df(3R)BSC321	96E6	96E9	0 (50) [a]
Df(3R)ED6232	96F10	97D2	0 (50) [a]
Df(3R)ED6255	97D2	97F1	0 (50) [a]
Df(3R)BSC497	97E6	98B5	0 (26)
Df(3R)BSC874	98E1	99A1	0 (10)
Df(3R)BSC501	98F10	99B9	0 (55)
Df(3R)BSC547	99B5	99C2	0 (97)
Df(3R)BSC620	99C5	99D3	0 (89)
Df(3R)Exel6214	99D5	99E2	0 (76)
Df(3R)BSC504	99F4	100A2	0 (41)
Df(3R)A113	100A	3Rt	0 (9)
Df(3R)BSC749	100B1	100C1	0 (29)
Df(3R)BSC505	100D1	100D2	0 (39)

Notes: [a]Approximate *n* based on number of vials scored, not individual flies scored. Some overlapping deficiencies are reported in Table 1.
