# Supplementary material for: unfulfilled Interacting Genes Display Branch-Specific Roles in the Development of Mushroom Body Axons in Drosophila melanogaster
Source: G3 (Bethesda). 2014 Feb 20;4(4):693–706. doi: 10.1534/g3.113.009829 (PMC4577660; doi:10.1534/g3.113.009829)
Supplement: Supporting Information [file supp_g3.113.009829_FileS1.pdf]

File S1

SUPPORTING METHODS

Candidate genes that were tested that are required for axon pathfinding and targeting include *α-Spectrin (α-Spec*^{lm88}) (Garbe and Bashaw 2007), *misshapen* (*msn*^{102}) (Ruan *et al.* 1999; Su *et al.* 2000), *spätzal* (*spz5*^{E03444}) (Zhu *et al.* 2008), *Kinesin-like protein at 64D* (*Klp64D*^{K1}) (Baqri *et al.* 2006; Ray *et al.* 1999; Sadananda *et al.* 2012), *veloren* (*velo*^{EY10127}) (Berdnik *et al.* 2012), *sugarless* (*sgl*^{08310}) (Cho *et al.* 2012), *Actin-related protein 3* (*Arp3*^{EP3640}) (Goncalves-Pimentel *et al.* 2011), *astray* (*aay*^{S042314}) (Salzberg *et al.* 1997), *RasGAP1* (*GAP1*^{B2}) (Yang and Terman 2012), *α-Tubulin67C* (*α-Tub67C*^{1}) (Wang *et al.* 2007), *tartan* (*trn*^{S064117}) (Kurusu *et al.* 2008), *capricious* (*caps*^{02937}) (Abrell and Jackle 2001), *commissureless* (*comm*^{M100380}) (Tear *et al.* 1996), *failed axon connections* (*fax*^{M7}, *fax*^{BG00833}, *fax*^{EY10882}, *fax*^{KG05016}), *Abl tyrosine kinase* (*Abl*^{2}) (Hill *et al.* 1995; Liebl *et al.* 2000), *schizo* (*siz*^{EY09677}) (Hummel *et al.* 1999), *chromosome bows* (*chb*^{4}) (Lee *et al.* 2004), *Tenascin major* (*Ten-m*^{05309}) (Hong *et al.* 2012; Mosca *et al.* 2012; Zheng *et al.* 2011), *Fps oncogene analog* (*Fps85D*^{X21}; also known as *Fer*) (Murray *et al.* 2006), *Tropomyosin 1* (*Tm1*^{02299}) (Stephan *et al.* 2012), *Specifically Rac1-associated protein 1* (*Sra1*^{EY06562}) (Bogdan *et al.* 2004), *hedgehog* (*hh*^{2}) (Huang and Kunes 1996; Salecker *et al.* 1998), and *axin* (*axn*^{EY10228}) (Chiang *et al.* 2009; Hida *et al.* 2012). *discs lost* (*dlt*^{04276}; also known as *DPATJ*), which shares a first untranslated exon with *α-Spec*, regulates photoreceptor morphogenesis and maintenance (Nam and Choi 2006; Pielage *et al.* 2003). Candidate genes that were already known to be involved in MB development include *Ptpmeg* (*Ptpmeg*^{1}) (Whited *et al.* 2007), *Ras homolog enriched in brain ortholog* (*Rheb*^{EY08085}) (Brown *et al.* 2012; Yaniv *et al.* 2012), *Tsc1* (*Tsc1*^{F01910}), *RPS6-p70-protein kinase* (*S6K*$^{l-1}$) (Yaniv *et al.* 2012), *tailless* (*tll*^{1}, *tll*^{149}) (Kurusu *et al.* 2009), and *tramtrak* (*ttk*^{le11}) (Nicolai *et al.* 2003). Another group of candidate genes are those involved in synaptic activity, learning and memory, or other MB-associated behaviors. These included *Shaker cognate b (Shab*^{MB02726}) (Gasque *et al.* 2005), *gryzun* (*gry*^{EY03013}) (Akalal *et al.* 2011; Dubnau *et al.* 2003), *dikar* (*dikar*^{d02315}) (Akalal *et al.* 2011), *Synaptotagminβ* (*Sytβ*^{PL00191}, *Sytβ*^{BG02150}) (Mackler and Reist 2001), *mushroom-body expressed* (*mub*^{04093}) (Grams and Korge 1998), *NMDA Receptor 1* (*NMDAR1*^{05616}) (Xia *et al.* 2005), *Synapse-associated protein 47kD* (*Sap47*^{EY07944}) (Reichmuth *et al.* 1995; Saumweber *et al.* 2011), *gilgamesh* (*gish*^{KG03891}) (Tan *et al.* 2010), *daughters against dpp* (*dad*^{J1E4}) (Rodal *et al.* 2011), *Syntaxin 1a* (*Syx1a*$^{\Delta 229}$) (Lagow *et al.* 2007; Wu *et al.* 1999), *jaguar* (*jar*^{1}) (Kisiel *et al.* 2011), *Syntaxin 18* (*Syx18*^{EY08095}) (Littleton 2000), *slowpoke* (*slo*^{1}) (Atkinson *et al.* 2000; Lee and Wu 2010), *Dopamine 1-like Receptor 2* (*DopR2*^{MB05108}; also known as *DAMB*) (Berry *et al.* 2012; Chen *et al.* 2012; Draper *et al.* 2007; Selcho *et al.* 2009; Seugnet *et al.* 2008), and *discs overgrown* (*dco*^{3}) (Yamazaki *et al.* 2007).
