# Supplementary material for: unfulfilled Interacting Genes Display Branch-Specific Roles in the Development of Mushroom Body Axons in Drosophila melanogaster
Source: G3 (Bethesda). 2014 Feb 20;4(4):693–706. doi: 10.1534/g3.113.009829 (PMC4577660; doi:10.1534/g3.113.009829)
Supplement: Supporting Information [file supp_g3.113.009829_TableS1.pdf]

Table S1 Deficiencies that fail to suppress the lethality induced by ectopic expression of *unfulfilled*

Deficiency	Start breakpoints	End breakpoints	Small eye flies (*n*)
Df(3L)ED50002	61A1	61B1	0 (31)
Df(3L)ED4177	61B1	61E2	0 (45)
Df(3L)BSC289	61F6	62A9	0 (46)
Df(3L)ED4191	61C3	62A2	0 (24)
Df(3L)BSC23	62E8	63B5-6	0 (78)
Df(3L)BSC671	63A2	63B11	0 (90)
Df(3L)BSC368	63F1	64A4	0 (91)
Df(3L)ED210	64B9	64C13	0 (167)
Df(3L)BSC884	64D6	64E7	0 (22)
Df(3L)BSC27	64D4-5	64E4-6	0 (134)
Df(3L)Exel8104	65F7	66A4	0 (37)
Df(3L)BSC375	66A3	66A19	0 (23)
Df(3L)BSC815	66C3	66D4	0 (39)
Df(3L)BSC389	66C12	66D8	0 (77)
Df(3L)ED4421	66D12	67B3	0 (43)
Df(3L)AC1	67A2	67D11-13	0 (14)
Df(3L)ED4457	67E2	68A7	0 (4)
Df(3L)ED4475	68C13	69B4	0 (72)
Df(3L)ED4486	69C4	69F6	0 (77)
Df(3L)ED4543	70C6	70F4	0 (131)
Df (3L)BSC774	71F1	72D10	0 (39)
Df(3L)ED4685	73D5	74E2	0 (50) [a]
Df(3L)ED4710	74D1	75B11	0 (50) [a]
Df(3L)BSC775	75A2	75E4	0 (50) [a]
Df(3L)BSC220	75F1	76A1	0 (50) [a]
Df(3L)ED229	76A1	76E1	0 (50) [a]
Df(3L)ED4858	76D3	77C1	0 (122)
Df(3L)BSC839	77B4	77C6	0 (86)
Df(3L)BSC797	77C3	78A1	0 (43)
Df(3L)BSC419	78C2	78D8	0 (31)
Df(3R)ED5147	82E7	83A1	0 (52)
Df(3R)ED5156	82F8	83A4	0 (47)
Df(3R)ED5177	83B4	83B6	0 (32)
Df(3R)BSC633	84B2	84C3	0 (91)
Df(3R)BSC466	84E1	85A10	0 (34)
Df(3R)ED5339	85D1	85D11	0 (64)
Df(3R)BSC476	85D16	85D24	0 (14)
Df(3R)BSC621	85F5	85F14	0 (52)
Df(3R)Exel7321	88A9	88B1	0 (79)
Df(3R)ED5664	88D1	88E3	0 (36)
Df(3R)BSC750	88E2	88E5	0 (39)
Df(3R)ED5705	88E12	89A5	0 (35)
Df(3R)BSC515	88F6	89A8	0 (31)
Df(3R)Exel7328	89A12	89B6	0 (23)
Df(3R)ED10642	89B17	89D5	0 (27)
Df(3R)Exel6270	89B18	89D8	0 (41)
Df(3R)BSC790	90B6	90E2	0 (86)
Df(3R)BSC819	93A2	93B8	0 (50)
Df(3R)Exel6272	93A4	93B13	0 (19)
Df(3R)ED10845	93B9	93D4	0 (22)
Df(3R)BSC677	93D1	93F14	0 (35)
Df(3R)ED6085	93F14	94B5	0 (24)
Df(3R)ED6096	94B5	94E7	0 (40)
Df(3R)BSC137	94F1	95A4	0 (35)
Df(3R)BSC489	94F3	95D1	0 (44)

Df(3R)Exel6196	95C12	95D8	0 (89)
Df(3R)ED6187	95D10	96A7	0 (42)
Df(3R)ED6220	96A7	96C3	0 (33)
Df(3R)BSC461	96B15	96D1	0 (89)
Df(3R)Exel6202	96D1	96D1	0 (50) [a]
Df(3R)Exel6203	96E2	96E6	0 (50) [a]
Df(3R)BSC321	96E6	96E9	0 (50) [a]
Df(3R)ED6232	96F10	97D2	0 (50) [a]
Df(3R)ED6255	97D2	97F1	0 (50) [a]
Df(3R)BSC497	97E6	98B5	0 (26)
Df(3R)BSC874	98E1	99A1	0 (10)
Df(3R)BSC501	98F10	99B9	0 (55)
Df(3R)BSC547	99B5	99C2	0 (97)
Df(3R)BSC620	99C5	99D3	0 (89)
Df(3R)Exel6214	99D5	99E2	0 (76)
Df(3R)BSC504	99F4	100A2	0 (41)
Df(3R)A113	100A	3Rt	0 (9)
Df(3R)BSC749	100B1	100C1	0 (29)
Df(3R)BSC505	100D1	100D2	0 (39)

Notes: [a]Approximate *n* based on number of vials scored, not individual flies scored. Some overlapping deficiencies are reported in Table 1.
